# Cell type-specific CLIP reveals that NOVA regulates cytoskeleton interactions in motoneurons

**DOI:** 10.1186/s13059-018-1493-2

**Published:** 2018-08-15

**Authors:** Yuan Yuan, Shirley Xie, Jennifer C. Darnell, Andrew J. Darnell, Yuhki Saito, Hemali Phatnani, Elisabeth A. Murphy, Chaolin Zhang, Tom Maniatis, Robert B. Darnell

**Affiliations:** 1 Laboratory of Molecular Neuro-Oncology, The Rockefeller University, 1230 York Ave., New York, NY 10065 USA; 2 Howard Hughes Medical Institute, The Rockefeller University, 1230 York Ave., New York, NY 10065 USA; 3 New York Genome Center, 101 Avenue of the Americas, New York, NY 10013 USA; 4 Department of Systems Biology, Columbia University, New York, NY 10032 USA; 5 Department of Biochemistry and Molecular Biophysics, Columbia University, New York, NY 10032 USA; 6 Center for Motor Neuron Biology and Disease, Columbia University, New York, NY 10032 USA

**Keywords:** Cell type-specific, CLIP, Motoneuron, NOVA, RNA, Alternative splicing, Alternative last exon usage, Septin

## Abstract

**Background:**

Alternative RNA processing plays an essential role in shaping cell identity and connectivity in the central nervous system. This is believed to involve differential regulation of RNA processing in various cell types. However, in vivo study of cell type-specific post-transcriptional regulation has been a challenge. Here, we describe a sensitive and stringent method combining genetics and CLIP (crosslinking and immunoprecipitation) to globally identify regulatory interactions between NOVA and RNA in the mouse spinal cord motoneurons.

**Results:**

We developed a means of undertaking motoneuron-specific CLIP to explore motoneuron-specific protein–RNA interactions relative to studies of the whole spinal cord in mouse. This allowed us to pinpoint differential RNA regulation specific to motoneurons, revealing a major role for NOVA in regulating cytoskeleton interactions in motoneurons. In particular, NOVA specifically promotes the palmitoylated isoform of the cytoskeleton protein Septin 8 in motoneurons, which enhances dendritic arborization.

**Conclusions:**

Our study demonstrates that cell type-specific RNA regulation is important for fine tuning motoneuron physiology and highlights the value of defining RNA processing regulation at single cell type resolution.

**Electronic supplementary material:**

The online version of this article (10.1186/s13059-018-1493-2) contains supplementary material, which is available to authorized users.

## Background

A thorough understanding of the complexities of the mammalian central nervous system (CNS) requires detailed knowledge of its cellular components at the molecular level. RNA regulation has a central role in establishing cell identity and function across the numerous cell types in the CNS [1–7]. Traditional whole tissue-based methods are particularly limited in their power to delineate cell type-specific RNA regulation in the mammalian CNS due to its vast cellular diversity and architectural complexity. While separation or induction of specific cell types in vitro provides a practical way for cell type-specific analyses [1–3, 8, 9], alteration of cellular biology due to loss of physiological contexts presents a significant caveat to this approach.

Recent technological breakthroughs using RiboTag and BAC-TRAP mouse lines have allowed for translational profiling at single cell type resolution [10–13]. These studies revealed remarkable differences in the population of translating mRNAs across various CNS cell types, highlighting the degree of molecular heterogeneity among neuronal cells. These methods offer important ways to study translated mRNAs in specific cell types, but do not provide a way to define other types of cell type-specific RNA regulation. Here we develop a complementary and more general means to study RNA processing and regulation in a cell type-specific manner.

RNA processing is regulated by RNA-binding proteins. Two of the best-studied are NOVA1 and NOVA2, neuron-specific KH-type RNA-binding proteins that bind to YCAY motifs and regulate alternative splicing and polyadenylation [14–16]. Using crosslinking and immunoprecipitation (CLIP), a method that allows stringent purification of protein–RNA complexes captured in vivo, we identified NOVA targets in mouse neocortex [16–19], and have estimated that NOVA participates in the regulation of ~ 7% of brain-specific alternative splicing events in mouse neocortex [20]. Interestingly, NOVA targets are specifically enriched for transcripts encoding proteins with synaptic functions—a group of transcripts that drives CNS cell type diversity [13, 15].

Indeed, NOVA proteins are essential for the function of multiple neuronal cell types [21–24]. In particular, we previously uncovered a pivotal role for NOVA in maintaining spinal motoneuron (MN) survival and physiology [5, 21, 25]. Here, to more precisely define NOVA-regulated RNA processing in spinal MNs, we developed a new strategy combining BAC-transgenic mice and CLIP to identify MN-specific RNA regulation. NOVA targets in MNs were especially enriched for genes encoding microtubule-, tubulin-, and cytoskeletal protein-binding proteins. These results led us to uncover a NOVA-mediated RNA processing event differentially regulated in MNs involving a cytoskeleton protein, Septin 8, which controls dendritic complexity. Cell type-specific CLIP revealed a previously unidentified role of NOVA in MNs, highlighting the importance of cell type-specific analysis of RNA regulation.

## Results

### BAC transgenic mice express epitope-tagged NOVA specifically in motoneurons

*Nova1* and *Nova2*, the two mammalian *Nova* paralogs, encode proteins harboring three nearly identical KH-type RNA binding domains. *Nova* genes are widely expressed among various neuronal cell types in the spinal cord, including the MNs. For NOVA2, two isoforms arise from alternative translational start sites, which do not shown detectable RNA binding differences (Yano Y. and Darnell R. B., unpublished data). Our strategy to study MN-specific NOVA regulation is twofold—to express AcGFP (*Aequorea coerulescens* green fluorescent protein)-tagged NOVA specifically in MNs, followed by CLIP using antibodies against the AcGFP epitope tag.

To test if an N-terminal AcGFP tag alters NOVA RNA binding specificity, we compared global RNA binding profiles of untagged and AcGFP-tagged long NOVA2 isoform. NIH/3T3 cells ectopically expressing NOVA2 or AcGFP-NOVA2 were subjected to HITS-CLIP using antibodies against NOVA and GFP, respectively (Additional file 1: Supplemental material and methods; Additional file 2: Figure S1A). We generated complex NOVA:RNA interactomes for both tagged and untagged NOVA2, as defined by 1,775,101 unique CLIP reads for NOVA2 and 9,778,818 for AcGFP-NOVA2. Both sets of CLIP data showed comparable genomic distributions (Additional file 2: Figure S1B). We identified NOVA2 and AcGFP-NOVA2 CLIP peaks, defined as regions with significantly higher CLIP tag coverage than gene-specific background expected from uniform random distribution [26, 27]. The canonical NOVA recognition motif, YCAY, was enriched around NOVA2 and AcGFP-tagged NOVA2 CLIP peaks to the same degree (Additional file 2: Figure S1C, D; R^2^ = 0.94), with tetramers that may overlap YCAY by three or more nucleotides (YCAY, NYCA, CAYN) showing the highest enrichment at both NOVA2 and AcGFP-NOVA2 peaks (red dots in Additional file 2: Figure S1D). These indicate that the N-terminal AcGFP tag does not perceptibly alter NOVA2 binding to its cognate RNA motifs.

**Fig. 1:**
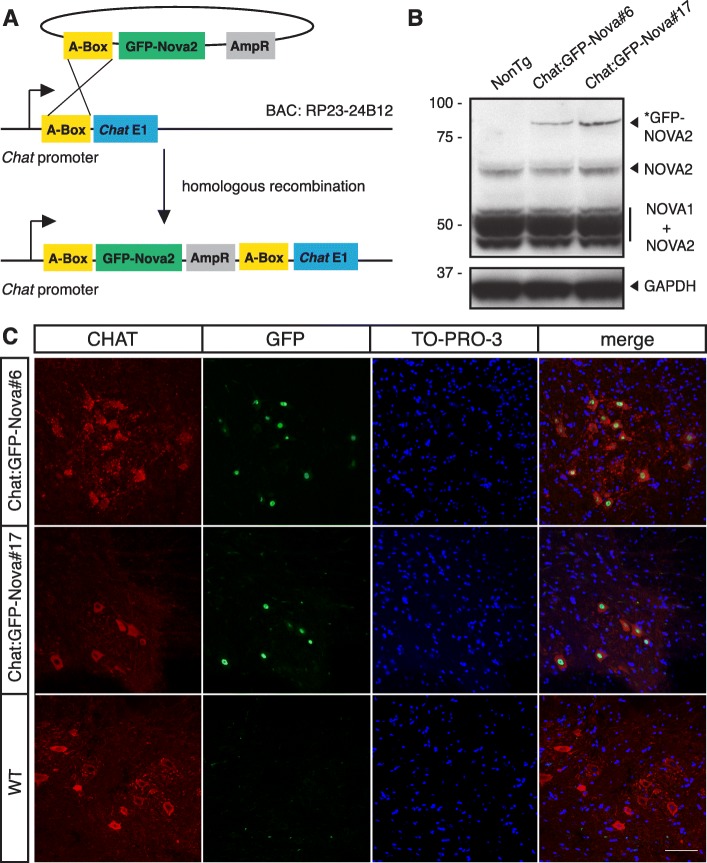
BAC-transgenic mouse lines express AcGFP-tagged NOVA2 in MNs. **a** The generation of recombinant BAC. A-box, a ~ 500-nt sequence homologous to the mouse *Chat* 5′ UTR region, mediated the insertion of a plasmid containing GFP-Nova2 coding sequence downstream of *Chat* promoter. **b** Western blot of spinal cord lysate from wild type (*WT*) mice and mice from Chat:GFP-Nova2 #6 and #17 lines using human anti-NOVA serum. The 93-kD GFP-NOVA2 was expressed only in transgenic spinal cords. GAPDH was blotted as a loading control. **c** Immunofluorescence on spinal cord transverse sections using antibodies against CHAT and GFP, and counterstained with TO-PRO-3. *Scale bar* represents 50 μm

Having confirmed the RNA binding fidelity of AcGFP-tagged NOVA2, we generated transgenic mice expressing AcGFP-tagged NOVA2 long isoform under the MN-specific choline acetyltransferase (*Chat*) promoter. The AcGFP-Nova2 cassette was inserted into a bacterial artificial chromosome (BAC) harboring the *Chat* promoter through homologous recombination (Fig. 1a) [28]. The engineered BAC was injected into fertilized C57BL/6 oocytes and two Chat:GFP-Nova2 BAC transgenic lines, #6 and #17, were selected and maintained from five original founder lines.

Chat:GFP-Nova2 transgenic mice were born at expected Mendelian ratios and were phenotypically indistinguishable from wild-type littermates throughout their lifespan (data not shown). Expression of the GFP-NOVA2 fusion protein was confirmed in the spinal cords of both Chat:GFP-Nova2 lines, but not the wild-type controls (Fig. 1b). To assess the MN-specific expression pattern of the transgene, we performed immunofluorescence staining on spinal cord transverse sections. GFP-NOVA2 expression was confined to CHAT-positive neurons in the transgenic lines (Fig. 1c). Thus, we have successfully established transgenic mouse lines with epitope-tagged NOVA specifically expressed in MNs.

### HITS-CLIP generates a robust transcriptome-wide NOVA–RNA interaction map in motoneurons

**Fig. 2:**
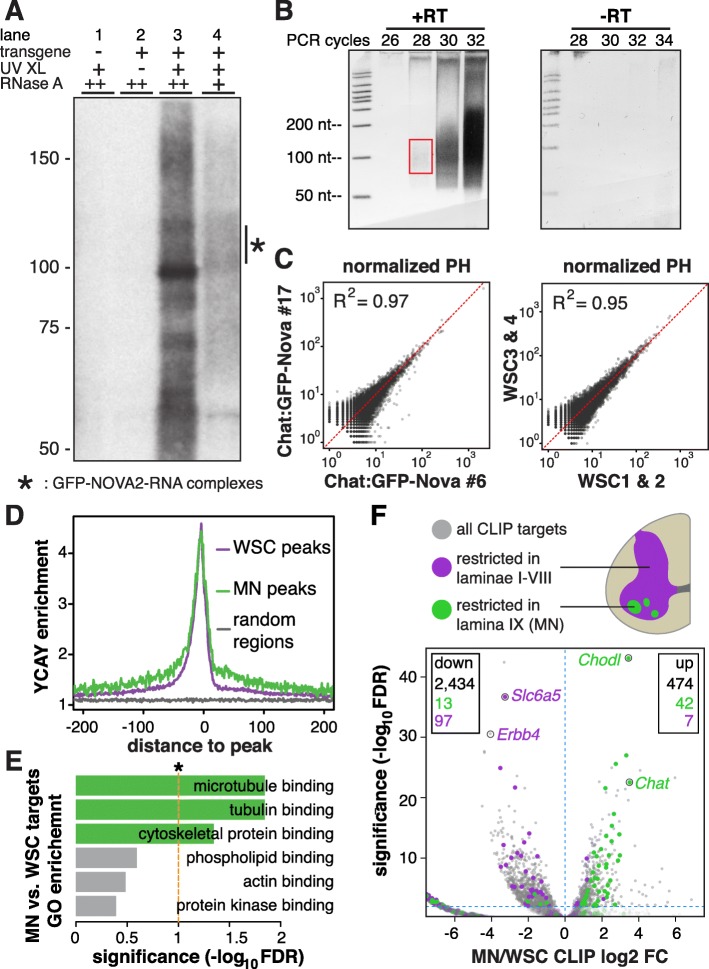
MN CLIP generated a bona fide and robust MN NOVA binding profile. **a** Autoradiogram showing NuPAGE separation of radio-labeled GFP-NOVA2–RNA complexes. Both the transgene and UV crosslinking were required for the presence of radio-labeled GFP-NOVA2–RNA complex (lanes 1–3), which appeared as a smear from 100 to 125 kD with partial RNase digestion and collapsed to a band around 95 kD in high RNase concentration (lanes 3 and 4). **b** Representative RT-PCR polyacrylamide gel images for CLIP library cloning. *Left panel* shows RT-PCR products at incremental PCR cycle numbers and *right panel* shows control reactions without reverse transcriptase. The *red box* indicates the cDNA (with 5′ and 3′ linkers) size range purified for subsequent cloning and sequencing. **c** Pairwise correlation of normalized CLIP peak height (*PH*) between #6 and #17 transgenic lines and between two WSC groups. Normalized PH is the sum of raw CLIP reads in a given peak normalized to the read depth of each individual experiment. For comparison purpose, CLIP reads in WSC groups were randomly downsampled in the same number of peaks (27,628) to match the complexity of MN CLIP. **d** Enrichment of YCAY around CLIP peaks. YCAY enrichment is calculated by normalizing the number of YCAY motifs starting at a given position relative to all WSC or MN CLIP peaks to the expected YCAY frequency based on random base distribution. **e** Molecular function GO enrichment of MNs relative to WSC NOVA targets. FDR values were calculated using hypergeometic test, followed by Benjamini-Hochberg multiple test correction. The top six GO terms with the smallest FDR values are shown. *Horizontal bars* shows significance of GO enrichment, with significantly enriched GO terms in *green*, and nonenriched in *gray*. *Dashed line* marks the FDR value 0.1. **f** Volcano plot of gene-wise CLIP read enrichment in MNs versus WSC. Illustration of spinal cord transverse section is shown on the *top right corner*, with Rexed laminae I–VIII in *purple* and lamina IX in *green*. Genes with a restricted expression pattern in Rexed lamina IX and laminae I–VIII, as curated by the Allen Spinal Cord Atlas, are shown as *green* and *purple dots*, respectively. All other NOVA CLIP targets are displayed as *gray circles*. Two MN and two interneuron marker genes are labeled. The numbers of NOVA CLIP targets with enriched or depleted NOVA binding in MNs are indicated at the upper right and left corners of the chart, respectively, with font colors indicating the Allen Spinal Cord Atlas curated subgroups. The *horizontal blue dashed line* denotes FDR value 0.01

To define NOVA binding sites in MNs, we undertook HITS-CLIP on Chat:GFP-Nova2 spinal cords using a mixture of two monoclonal antibodies against GFP to maximize avidity and specificity. GFP immunoprecipitation was performed using wild-type or transgenic spinal cords with or without UV crosslinking, followed by ^32^P-labeling of bound RNA. The presence of labeled GFP-NOVA2–RNA complexes was dependent on both UV crosslinking and the expression from the Chat:GFP-Nova2 transgene, further demonstrating the specificity of GFP-NOVA2 CLIP under these conditions (Fig. 2a, lanes 1–3). Partially digested RNA crosslinked to GFP-NOVA2 was subjected to subsequent library preparation steps (Fig. 2a, lane 4; “Methods”). Importantly, the production of cDNA was dependent on reverse transcriptase (Fig. 2b), confirming the absence of DNA contaminants in the CLIP RNA libraries. cDNA inserts between 30 and 80 nucleotides (nt; Fig. 2b) were selected for next-generation sequencing (NGS) and further analysis.

Four sets of biological replicate HITS-CLIP experiments were performed on each Chat:GFP-Nova2 transgenic line, with each biological replicate consisting of pooled spinal cord samples from five to seven 3-month-old mice. We obtained a total of 2,023,726 unique CLIP reads from these eight biological replicates. Since AcGFP-Nova2 expression is confined to MNs, we refer to this group of CLIP reads as MN NOVA CLIP reads. As a reference to all NOVA binding sites in the whole spinal cord, we additionally performed four standard NOVA CLIP experiments on endogenous NOVA1 and NOVA2 in 3-month-old wild-type mouse whole spinal cords (WSC), generating 17,353,049 unique WSC NOVA CLIP reads (Additional file 2: Figure S2A; Additional file 3A). CLIP reads from MN CLIP showed higher intronic and less exonic distribution compared to those from WSC CLIP (*p* = 4.2 × 10^− 7^; Additional file 2: Figure S2B).

NOVA peaks in MN and WSC CLIP datasets were defined separately [27]. A total of 25,681 CLIP peaks (*p* ≤ 0.01) were identified for MN NOVA CLIP, which harbor CLIP reads originating from at least four of the eight biological replicates (biologic complexity (BC) ≥ 4 out of 8; Additional file 3 B) [16]. In parallel, we identified 218,794 WSC NOVA peaks represented in at least two out of four biological replicates (BC ≥ 2 out of 4; Additional file 3C). The CLIP data from our two transgenic lines was highly correlated (R^2^ = 0.97), as were WSC CLIP reads (Fig. 2c), underscoring the ability of cell type-specific HITS-CLIP to reproducibly and quantitatively measure in vivo cellular protein–RNA interactions.

MN HITS-CLIP generated a genome-wide binding profile characteristic of endogenous NOVA proteins. The canonical NOVA binding motif YCAY was significantly and similarly enriched around MN and WSC CLIP peaks (Fig. 2d). WSC NOVA CLIP peaks mapped to 12,445 mm9-annotated Entrez genes, including to the alternative spliced regions of 303 out of 335 known NOVA-regulated genes (Additional file 3D); MN CLIP peaks mapped to 4450 Entrez genes, including to 174 known NOVA-regulated alternatively spliced regions (Additional file 3E) [20]. It is of note that when compared to all NOVA-regulated genes in the WSC, this subset of MN NOVA targets are especially enriched for genes encoding microtubule-binding (GO:0008017), tubulin-binding (GO:0015631), and cytoskeletal protein-binding proteins (GO:0008092) (hypergeometric test, false discovery rate (FDR) < 0.05; Fig. 2e and Additional file 3F), suggesting specialized functions for NOVA–RNA regulation in MNs.

Since transcript abundance directly affects CLIP signal intensities, we expected to see higher MN/WSC CLIP signals on MN-enriched transcripts. Therefore, we tested whether transcripts known to be enriched in MN, when compared with WSC, showed enrichment of NOVA binding in MN NOVA CLIP. The numbers of WSC or MN CLIP reads within respective peaks were summed for each gene, followed by analysis using the Bioconductor edgeR package [29] to identify genes with differential enrichment of CLIP reads in MN or WSC; 474 and 2434 transcripts showed enriched and depleted NOVA binding in MN compared to WSC, respectively (FDR ≤ 0.01; Fig. 2f and Additional file 3G, H). Consistent with the cell type specificity of GFP-NOVA2 CLIP, the known MN markers *Chat* and *Chodl* were among the top transcripts with the most significantly enriched GFP-NOVA2 CLIP reads coverage (FDR = 2.61 × 10^− 23^ and 8.39 × 10^− 44^, respectively; Fig. 2f) [30]. Conversely, *Slc6a5*, a glycine transporter gene known as an inhibitory neuron marker [31], as well as *Erbb4*, an interneuron-restricted receptor tyrosine kinase [32, 33], were two transcripts with the most significantly depleted MN CLIP reads coverage (FDR = 1.84 × 10^− 37^ and 2.31 × 10^− 31^, respectively, Fig. 2f).

We further systematically examined transcripts with well-defined anatomic expression patterns in spinal cord. Spinal cord gray matter exhibits a pattern of lamination consisting of ten laminal layers, with large alpha-MN pools located in lamina IX [34]. Based on the Allen Spinal Cord Atlas generated and curated in situ hybridization data [35], 166 transcripts are exclusively expressed in lamina IX, of which 55 displayed differential NOVA binding (FDR ≤ 0.01) in MN. Meanwhile, 301 transcripts are restricted in one or more laminae other than lamina IX, of which 104 showed significant differential NOVA binding (FDR ≤ 0.01). Among this group of 159 (55 + 104) transcripts with differential NOVA binding in MN, the enrichment or depletion of NOVA CLIP signals on these transcripts in MN compared to WSC were highly concordant with transcript spatial expression patterns (green and purple dots in Fig. 2f), in that 42 of the 55 (76%) lamina IX enriched transcripts had enriched NOVA binding in MN (*p* < 2.2 × 10^− 16^, chi-squared test), and 97 out of the 104 (93%) lamina I–VIII enriched transcripts had lower NOVA binding in MN (*p* = 0.007, chi-squared test). Taken together, we conclude that HITS-CLIP in Chat:GFP-Nova2 lines captured MN-specific transcriptome-wide NOVA–RNA interactions.

### NOVA displays MN-specific binding patterns

We were particularly interested in NOVA binding sites along a given gene that were disproportionally over- or under-represented in MNs compared to WSC (Additional file 2: Figure S3). To identify such NOVA–RNA interactions, we bioinformatically pooled WSC and MN CLIP reads to define peak regions referred as “joint peaks” (JPs), and grouped these JPs by genes (Additional file 2: Figure S3 and “Methods”). For each gene with two or more JPs, we performed pairwise Fisher’s exact test to identify JPs with disproportionally enriched MN or WSC CLIP reads (Additional file 2: Figure S3). This subset of JPs denotes NOVA binding sites over- or under-represented in MN compared to WSC.

**Fig. 3:**
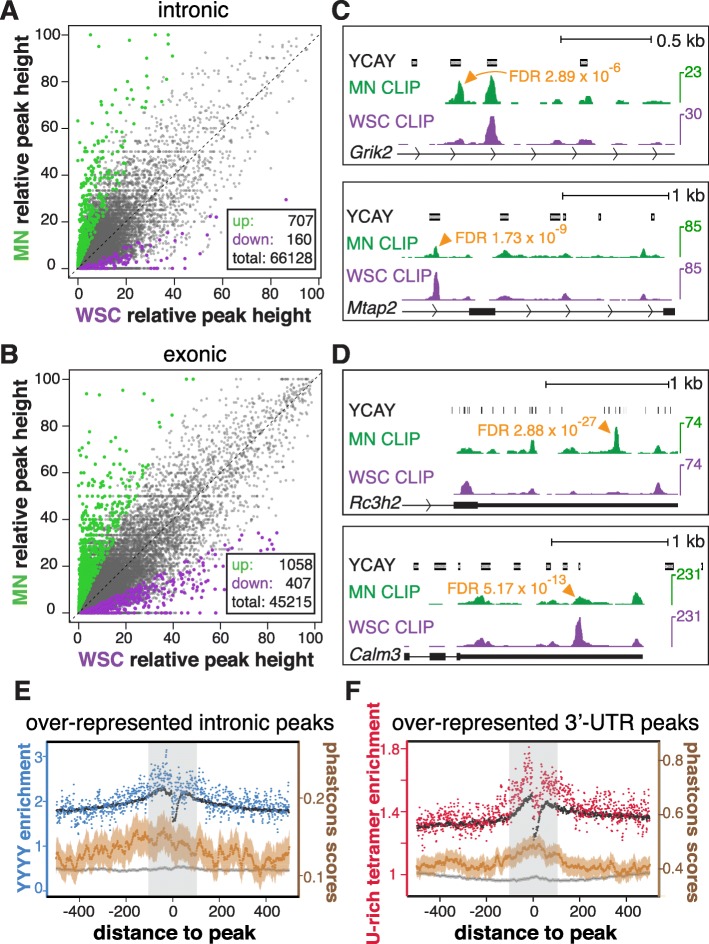
NOVA displays MN-specific binding patterns. **a, b** Pairwise comparison of intronic and exonic relative NOVA peak heights in MNs and WSC using the method illustrated in Additional file 2:Figure S3. Relative NOVA peak heights in introns or exons are calculated as following: 100 × Number of MN or WSC CLIP reads in a JP/Number of MN or WSC CLIP reads in all intronic or exonic JPs in the corresponding gene. NOVA peaks disproportionally over- or under-represented in MN (FDR ≤ 0.1, fold change ≥ 2) are shown in *green* and *purple*, respectively. The numbers of differential peaks and total peaks with sufficient coverage for the analysis are shown in the *boxes*. **c, d** UCSC genome browser images illustrating disproportionally different intronic (**c**) an exonic (**d**) NOVA binding sites in MNs. The YCAY track demarcates clusters of NOVA binding motifs. The WSC and MN CLIP tracks are pooled HITS-CLIP results, normalized for the displayed regions so that the highest unchanged peaks in WSC and MN share the same height. Significantly different NOVA binding sites between MNs and WSC are marked by *arrowheads* with FDR values indicated. UCSC gene annotation and transcript direction are shown at the bottom of each panel. **e, f** Positional enrichment of YYYY and U-rich tetramers and phastcons scores around intronic (**e**) and 3′ UTR (**f**) NOVA peaks over-represented in MNs, respectively. YYYY and U-rich tetramer enrichment is calculated from motif frequencies at each base position relative to over-represented (*blue* or *red*) or all (*black*) MN CLIP peaks in introns or 3′ UTRs, normalized by their expected frequencies based on random base distribution. Phastcons scores are plotted with *solid dots* denoting the mean phastcons values at a given base position, and *lighter lines* denoting 95% confidence intervals. *Dark gray* and *brown* represent phastcons scores around all and over-represented intronic/3′ UTR NOVA peaks, respectively. Motif enrichment scales are on the *left* and phastcons score scales are on the *right*. *Light gray boxes* highlight regions 100 nt around NOVA peaks

Using this method, we identified 121,216 genic JPs after performing our peak-finding algorithm on pooled WSC and MN CLIP reads and filtering for biological reproducibility (Additional file 2: Figure S3). Analysis of intronic and exonic JPs as two separate groups revealed 707 (1.07%) over- and 160 (0.24%) under-represented intronic JPs in MNs (Fig. 3a and Additional file 4A), as well as 1058 (2.34%) over- and 407 (0.90%) under-represented exonic JPs in MNs (FDR ≤ 0.1, fold change ≥ 2; Fig. 3b and Additional file 4B). The AcGFP epitope tag does not significantly account for the observed NOVA binding differences between MN and WSC (Additional file 2: Figure S4A–C and Fig. 3a, b). Examples of disproportionally over- and under-represented NOVA binding sites are shown in Fig. 3c (intronic) and 3d (exonic).

We examined potential RNA sequence signatures around MN-specific NOVA binding sites (FDR ≤ 0.1, fold change ≥ 2) by analyzing neighborhood tetramer distributions. We discovered a marked overrepresentation of polypyrimidine (YYYY) tetramers around intronic NOVA binding sites over-represented in MN (Fig. 3e), as well as a striking enrichment of U-rich (two or more uridines) tetramers around 3′-UTR NOVA binding sites over-represented in MN (Fig. 3f). For both polypyrimidine and U-rich tetramers, differential enrichment is confined to regions 100 nt up- and downstream of over-represented MN peaks (Fig. 3e, f). Interestingly, regions flanking changed MN peaks are evolutionarily more conserved compared to all intronic or 3′-UTR NOVA peaks (Fig. 3e, f), suggesting the potential functional importance of differential NOVA binding sites in MN. Taken together, these data suggest that a large number of binding sites are differentially bound by NOVA independent of potential transcript level variations between WSC and MN, such that NOVA displays MN-specific binding patterns.

### MN-specific NOVA binding predicts MN-specific alternative splicing

Since NOVA plays an important role in regulating neuronal transcript splicing, we tested whether MN-specific NOVA binding correlates with MN-specific alternative splicing. We took advantage of a high quality MN RNA-seq dataset, where spinal MNs in 3-month-old wild-type mice were collected by laser capture microdissection (LCM) and subjected to RNA-seq [36]. For the WSC transcriptome, we performed RNA-seq on age-, genotype-, and strain background-matched whole spinal cords. Approximately 36 and 80 million mappable reads were obtained from either biological replicates of MN and WSC RNA-seq, respectively, and alternative splicing analysis was performed as described [26] (http://zhanglab.c2b2.columbia.edu/index.php/Quantas).

**Fig. 4:**
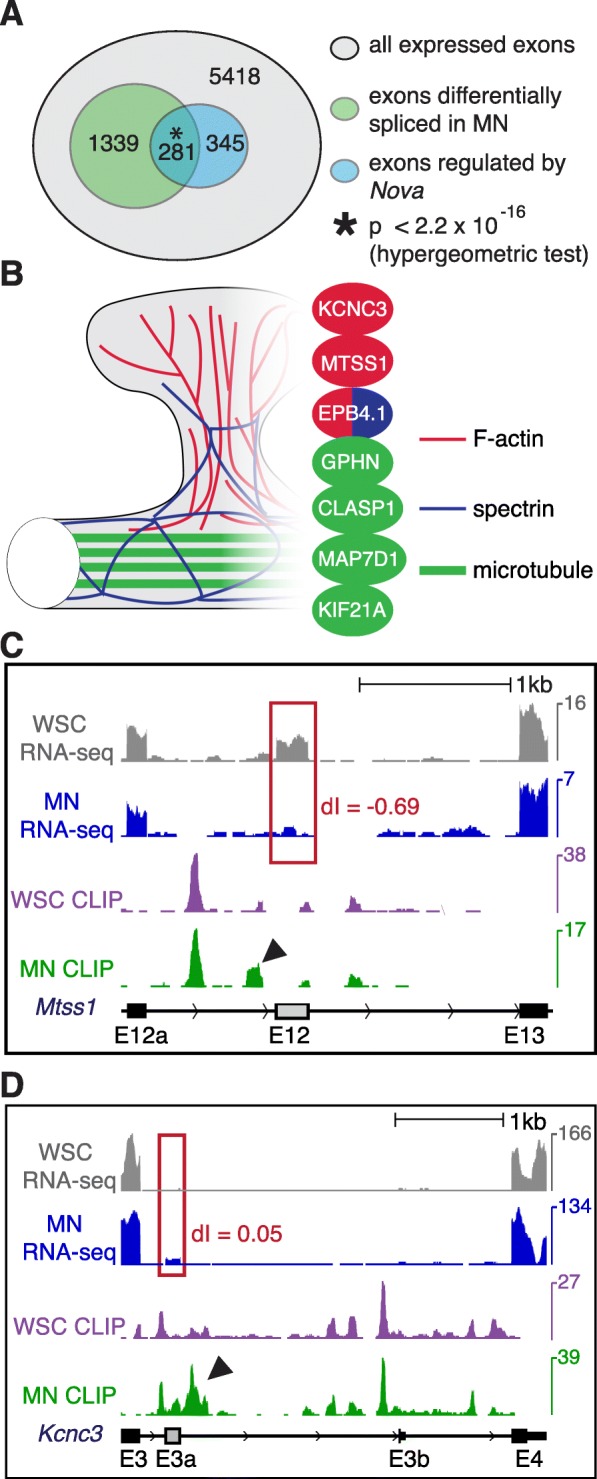
MN-specific NOVA binding correlates with MN-specific alternative splicing. **a** Venn diagram showing overlap between cassette exons differentially spliced in MN and known *Nova* targets [20] among all expressed alternative exons (see “Methods”). *P* value is calculated by hypergeometric test. **b** Illustration of cytoskeleton structures in part of a dendrite and a dendritic spine. Actin filaments are represented in *red*, microtubules in *green*, and spectrin in *navy*. Differentially regulated MN NOVA targets are represented in colors corresponding to their interacting cytoskeletal component(s). **c, d** UCSC genome browser images illustrating correlation between differential NOVA binding and MN-specific alternative splicing in the cases of *Mtss1* E12 (**c**) and *Kcnc3* E3a (**d**). The YCAY track demarcates clusters of Nova-binding motifs. WSC and MN RNA-seq tracks are RNA-seq results from 3-month-old whole spinal cord (this study) and laser dissected motoneurons [36], respectively, with biological replicates pooled. For alternative splicing visualization, these two tracks share the same maximum heights of flanking exons. Exons differentially spliced (FDR ≤ 0.1) in MNs versus WSC are highlighted in *red boxes*. The WSC and MN CLIP tracks are pooled HITS-CLIP results, normalized for the given regions so that the highest unchanged peaks in WSC and MN share the same height. Read coverage of RNA-seq and CLIP are scaled on the right axes from zero to the indicated reads per million. Significantly different NOVA binding sites between MNs and WSC (FDR ≤ 0.1) are marked by *arrowheads*. UCSC gene annotation and transcript direction are shown at the bottom of each panel, with alternative exons marked in *gray*. For *Mtss1* E12, an increase of the NOVA peak (*arrowhead*) immediately upstream in MNs correlates with increased E12b inclusion in MNs. Similarly, a dramatic increase of NOVA binding immediately downstream of *Kcnc3* E3a (*arrowhead*) correlates with activated E3a splicing in MNs

Around 30% (1620 out of 5418) of MN-expressed alternative exons showed biologically consistent differential splicing in MNs compared to WSC (FDR ≤ 0.1, BC = 2 out of 2; Fig. 4a and Additional file 5A). Strikingly, an even higher proportion of MN-expressed *Nova* targets are differentially spliced between MNs and WSC (281 out of 626, 45%, FDR ≤ 0.1, BC2 out of 2; Fig. 4a). The significant enrichment of *Nova* targets among exons differentially spliced in MNs (*p* < 2.2 × 10^− 16^, hypergeometric test) suggests that *Nova* contributes in an important way to MN-specific splicing patterns.

We tested whether MN-specific NOVA binding correlated with MN-specific alternative splicing. Although NOVA binding in broader regions may influence splice site choice, high confidence predictions on whether NOVA promotes or represses alternative exons is achieved when NOVA binds within a window of 400 nt around the regulated exons [16]—upstream binding highly correlates with splicing repression, and downstream binding with splicing activation. Transcriptome wide, 13 NOVA binding sites over- or under-represented in MNs (FDR ≤ 0.1) are located in these regulatory “hotspots” around alternative exons with RNA-seq coverage sufficient for analysis. Interestingly, the majority of these exons (9 out of 13, 69%) showed MN-specific splicing patterns (FDR ≤ 0.1). Based on our NOVA–RNA map, differential NOVA binding patterns in MNs compared to WSC correctly predicted increase or decrease of alternative exon inclusion in MNs compared to WSC for eight out of the nine alternative exons (89%) (Additional file 5B; Fig. 4c, d; Additional file 2: Figure S5), consistent with NOVA mediating a direct action to regulate MN-specific alternative splicing.

Intriguingly, the seven genes hosting these nine MN-specific splicing events encode a functionally coherent set of proteins. Six of the seven gene products, i.e., MTSS1, MAP7D1, KIF21A, EPB4.1|3, CLASP1, GPHN, and KCNC3, are known to interact with cytoskeleton components (Fig. 4b) [37–43]. For example, MTSS1 binds to actin monomers and induces membrane protrusion [37, 44]. *Mtss1* harbors two alternatively spliced exons, E12 and E12a, at the 3′ end of its coding sequence, and inclusion of E12a, but not E12, is necessary to promote neuritogenesis [45, 46]. Interestingly, MN CLIP revealed a unique NOVA binding site 109 nt upstream of *Mtss1* E12 in MN (threefold increase in relative peak height; FDR = 0.046; Fig. 4c), which would predict based on the NOVA-RNA map that NOVA binding would inhibit E12 in MNs. Indeed, we observed a remarkably lower E12 inclusion rate in MNs compared to WSC (dI (MN-WSC) = − 0.69, FDR = 9.65 × 10^− 7^; Fig. 4c).

Another example is *Kcnc3*, which encodes a pan-neuronal voltage-gated potassium channel Kv3.3 [47, 48]. MN CLIP and RNA-seq revealed a new alternative exon E3a which showed a significantly higher inclusion rate in MNs compared to WSC (dI (MN-WSC) = 0.05, FDR = 1.38 × 10^− 14^). The increased utilization of E3a in MNs positively correlated with a dramatically enhanced NOVA binding site in MN 121 nt downstream of E3a (fourfold increase, FDR = 4.02 × 10^− 7^; Fig. 4d). Interestingly, inclusion of E3a would lead to a Kv3.3 isoform with an extended C-terminal proline-rich domain, which has been shown to modulate channel inactivation through triggering actin nucleation at the plasma membrane [43]. Taken together, these observations suggest that unique NOVA binding patterns around alternative exons in MNs contribute to MN-specific biology, particularly in shaping the cytoskeleton and regulating cytoskeleton interactions in unique ways within spinal cord MNs.

### NOVA differentially regulates *Sept8* alternative last exon usage in MNs

**Fig. 5:**
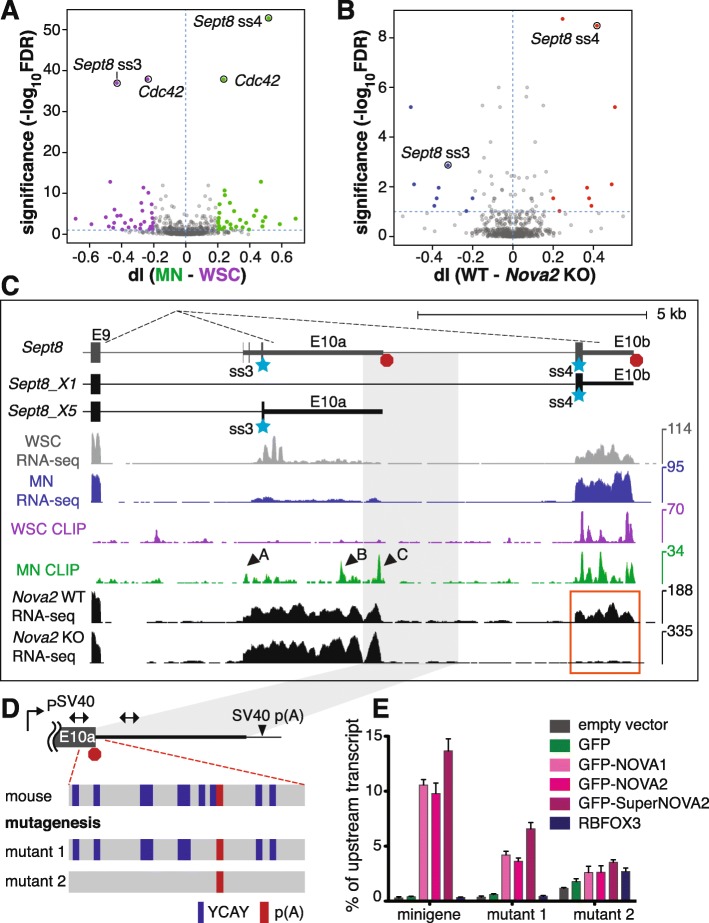
NOVA promotes *Sept8* exon 10b usage in MNs. **a, b** Volcano plot of differential ALE usage in MNs versus WSC (**a**) and *Nova2* wild type (*WT*) versus KO mouse brains (**b**). ALEs with higher inclusion rates in MNs and *Nova2* WT (dI ≥ 0.2, FDR ≤ 0.1) are labeled in *green* and *red*, respectively. ALEs with lower inclusion rates in MNs and *Nova2* WT (dI ≤ 0.2, FDR ≤ 0.1) are labeled in *purple* and *blue*, respectively. The *blue horizontal dashed lines* denote FDR value 0.1. **c** UCSC genome browser images illustrating the correlation between differential NOVA binding pattern and *Sept8* ALE usage in MNs. Partial gene and transcript structures of *Sept8* are shown on top. Alternative last exons 10a and 10b are utilized in the X5 and X1 isoforms, respectively. *Blue stars* mark the two predominant alternative 3′ splice sites used in the adult spinal cord. *Red octagons* mark polyadenylation sites. For WSC and MN RNA-seq and NOVA CLIP tracks, see Fig. 4c, d legend for reference. *Arrowheads* mark significantly over-represented NOVA binding sites in MNs. E18.5 WT and *Nova2* knockout mouse brain RNA-seq are displayed in *black*. Inclusion of exon 10b is dependent on NOVA, as highlighted by the *orange box*. *Light gray box* marks the genomic region included in the Sept8 minigene in **d**. **d** The Sept8 minigene construct. The 3′ end of *Sept8* exon 10a and its adjacent intronic sequence were inserted downstream of the SV40 promoter and upstream of the SV40 poly(A) signal. *Double-arrowed segments* represent qPCR amplicons used for measuring transcription readthrough. The region surrounding the poly(A) signal is enlarged, with YCAY motifs marked in *navy* and the poly(A) signal in *red*. Mutant 1 minigene lacks the two YCAY motifs proximal to the poly(A) signal. The mutant 2 minigene is devoid of YCAY in the 150-nt region surrounding the poly(A) signal. **e** Sept8 minigene assay. COS-1 cells expressing minigene variants and indicated proteins were harvested for RNA isolation and qRT-PCR analysis. Two amplicons illustrated in Fig. 5d were used to measure RNA levels up- and downstream of exon 10a poly(A) sites, respectively. Y-axis represents percentage of downstream relative to upstream transcript level. *Error bars* represent standard error of the mean (SEM) based on three independent replicates

Regulation of alternative last exon (ALE) usage involves intricate interplay between splicing and cleavage/polyadenylation, two processes both regulated by NOVA [16]. We therefore investigated the potential role that NOVA played in regulating ALE usage in MNs. Sixty-five ALEs in 34 genes were differentially included in MNs compared to WSC (FDR ≤ 0.1, |dI| ≥ 0.2; Fig. 5a and Additional file 6A), with the top differentially utilized ALEs residing in two functionally related genes, *Sept8* and *Cdc42* (Fig. 5a) [49–51]. We also examined the published *Nova2* wild-type and knockout (KO) mouse brain RNA-seq dataset [24], and identified additional NOVA regulation on 17 ALEs in 9 genes (FDR ≤ 0.1, |dI| ≥ 0.2, Fig. 5B and Additional file 6 B). Interestingly, among all genes with ALEs, *Sept8* was the only NOVA target differentially regulated in MNs compared to WSC.

*Sept8* encodes a family member of the septin proteins, which are multi-functional components of the cytoskeleton [52, 53]. In neurons, septins regulate dendritic and axon morphology through modulating actin and microtubule dynamics [54–56]. Although much is known about other septins, SEPT8 is a more recently described family member and less well characterized. Mouse *Sept8* harbors two alternative terminal exons, exon 10a and 10b (Fig. 5c). Four mutually exclusive splice acceptors in *Sept8* exons 10a and 10b can directly join downstream of exon 9, with splice acceptors 3 in exon 10a and 4 in exon 10b utilized in > 85% of *Sept8* transcripts in adult mouse spinal cords (Fig. 5c). Comparison of ALE usage altered between *Nova2* wild-type and KO mouse brains [24] showed markedly lower inclusion rate for exon 10b in *Nova2* KO (dI = 0.42, FDR = 3.32 × 10^− 9^; Fig. 5c), suggesting that NOVA promotes splice acceptor 4 usage, exon 10a exclusion, and exon 10b inclusion (Fig. 5c). In MNs, RNA-Seq data indicate that the majority of *Sept8* transcripts use splice acceptor 4 (65% in MNs vs 22% in WSC), while splice acceptor 3 is preferentially bypassed (20% in MNs vs 72% in WSC; Fig. 5c). This observed differential ALE usage between MNs and WSC coincides with higher NOVA binding in MNs at three binding sites located in exon 10a (site A fold change = 10, FDR = 0.0050; site B fold change = 3.4, FDR = 0.0046; site C fold change = 4.4, FDR = 3.84 × 10^− 5^; Fig. 5c). NOVA binding in site C is in a highly conserved YCAY-dense region encompassing the putative polyadenylation site at the 3′ end of exon 10a (Fig. 5d and Additional file 2: Figure S6A).

NOVA has been shown to prevent cleavage/polyadenylation through binding in close proximity to polyadenylation sites [16]. We tested whether NOVA association with the 3′ end of *Sept8* exon 10a blocked cleavage/polyadenylation, thus allowing for RNA polymerase II (PolII) readthrough and inclusion of the downstream exon 10b. A minigene was constructed using the genomic region of the 3′ end of exon 10a and the 5′ part of intron 10 (Fig. 5d). We co-transfected this minigene reporter with vectors expressing NOVA proteins or controls into COS-1 cells (Additional file 2: Figure S6B), which lack endogenous NOVA, followed by quantification of RNA levels up- and downstream of the E10a polyadenylation site. In the absence of NOVA proteins, transcription terminated efficiently at the E10a polyadenylation site, as measured by the less than 0.5% transcription readthrough rate (Fig. 5e). Exogenous NOVA proteins increased the downstream readthrough dramatically (> 10%, > 25-fold; Fig. 5e), while another RNA-binding protein (RBFOX3) showed no effect on cleavage/polyadenylation at E10a (Fig. 5e), indicating that NOVA proteins efficiently blocked cleavage/polyadenylation and promoted downstream transcription.

To test whether direct NOVA association with the YCAY sites is necessary for the observed regulation, we generated two mutant minigenes by disrupting YCAY sites while preserving the GC content (Fig. 5d). Mutant 1, with the two YCAY sites proximal to the E10a poly(A) site mutated, showed moderately dampened responses (6–11-fold) to NOVA overexpression (Fig. 5e). In contrast, little NOVA regulation (less than twofold; Fig. 5e) was observed for mutant 2, where all YCAY sites within 150 nt of the poly(A) site were disrupted. Taken together, these results suggest that NOVA promotes *Sept8* exon 10b inclusion by binding close to the polyadenylation site in exon 10a and boosting readthrough transcription and utilization of exon 10b, and that strengthened NOVA binding around the exon 10a poly(A) site in MNs led to higher exon 10b usage observed in MNs.

### The *Sept8* exon specifically promoted by NOVA in MNs encodes a palmitoylated filopodia-inducing motif that enhances dendritic arborization

**Fig. 6:**
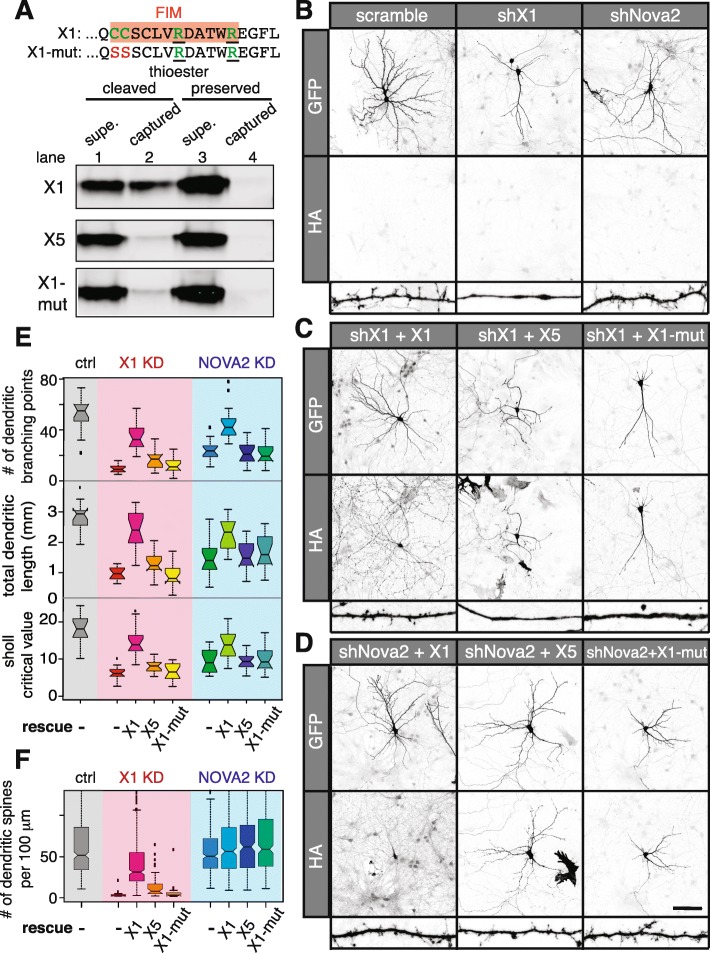
NOVA-dependent SEPT8 isoform promotes dendritic arborization and spine morphogenesis. **a** Detection of palmitoylated SEPT8-X1 by acyl-RAC (as illustrated in Additional file 2:Figure S7A). *Top*: C-terminal amino acid sequence of SEPT8-X1. *Orange box* highlights the FIM, with *green letters* marking palmitoylated cysteines and nearby basic amino acid residues. C469 and C470 were mutated to serine residues in SEPT8-X1-mut. *Bottom*: COS-1 cells expressing HA-tagged SEPT8 variants were used for the acyl-RAC assay. Immunoblotting of supernatant and thiopropyl-sepharose captured fractions using an antibody against HA is shown. Ten percent of supernatants were loaded compared to the captured fractions. **b–d** Representative maximum projected confocal images of hippocampal neurons expressing shRNAs and HA-tagged SEPT8 or controls. GFP, which is co-expressed from the shRNA constructs, is used as a neurite tracer. Anti-HA labels exogenously expressed SEPT8 variants. Representative images of dendrites are shown at the bottom of each panel. *Scale bar* represents 100 μm. **e, f** Boxplots evaluating dendritic arbor complexity (**e**) and dendritic spine density (**f**) in neurons with SEPT8-X1 or NOVA2 KD and SEPT8 rescue. Number of dendritic branching points, total dendritic lengths, and sholl analysis critical values are plotted in **e**, and number of dendritic spines per 100 μm is plotted in **f**. Measurements in neurons expressing the control, shX1, and shNova2 shRNAs are highlighted by the *gray*, *pink*, and *blue boxes*, respectively. Quantification was based on 15–17 neurons per group

Alternative usage of exons 10a and 10b confers the C-terminal variation between SEPT8 protein isoforms X5 and X1, respectively. SEPT8 undergoes palmitoylation in vivo [57], which is a reversible post-translational process of attaching a 16-carbon saturated fatty acid to cysteine residues [58]. Although the definitive palmitoylation site(s) in SEPT8 is unknown, the only predicted sites are two exon 10b-encoded cysteine residues (C469, C470) in the NOVA-promoted SEPT8-X1 isoform [59]. To assess whether C469 and C470 mediate SEPT8-X1 palmitoylation, we expressed SEPT8 isoforms in COS-1 cells and employed the acyl-resin-assisted-capture (acyl-RAC) assay for palmitoylation detection [60, 61]. This assay relies on the indirect capture of palmitoylated proteins facilitated by palmitoyl-ester-specific cleavage (Additional file 2: Figure S7A). Whereas acyl-RAC failed to detect any palmitoylated SEPT8-X5, 5–10% of SEPT8-X1 was shown to be palmitoylated, as evidenced by the presence of SEPT8-X1 in the resin captured fraction (Fig. 6a, lane 2). This capture was dependent on palmitoyl-ester-specific cleavage (Fig. 6a, lanes 3 and 4). Mutations in C469 and C470 (SEPT8-X1-mut, C469S/C470S) prevented the detection of SEPT8-X1 palmitoylation (Fig. 6a), indicating that the two cysteines encoded by the NOVA-promoted exon 10b are the sites for palmitoyl addition. The data here collectively suggest that NOVA-regulated alternative RNA processing in MNs mediates isoform-specific SEPT8 palmitoylation.

Interestingly, we discovered that *Sept8* exon 10b encodes a potential filopodia-inducing motif (FIM), characterized by two adjacent palmitoylated cysteines (C469, C470) and nearby basic residues (R475, R480) (Fig. 6a, orange box) [62]. It has been shown that the palmitoylated FIM was sufficient to promote dendritic branching and spine formation in neurons [62]. To assay the functional effects of SEPT8-X1 on dendritic morphology, we performed isoform-specific knockdown (KD) using a construct co-expressing GFP and an shRNA targeting *Sept8* exon 10b (shX1) in primary mouse hippocampal neurons which express SEPT8-X1 (Additional file 2: Figure S7B). KD efficiency (79%) and isoform specificity of shX1 was demonstrated in COS-1 cells expressing exogenous SEPT8-X1 and X5 (Additional file 2: Figure S7C). Compared to neurons expressing the control scramble shRNA, neurons transfected with shX1 had significantly less complex dendritic arbors, as indicated by an 82% reduction in the number of dendritic branching points (Fig. 6b, e; *p* = 2.9 × 10^− 9^). Meanwhile, total dendritic length showed a 65% reduction (*p* = 9.2 × 10^− 11^), and sholl analysis revealed a similar 66% reduction in critical value in neurons with SEPT8-X1 KD (Fig. 6e; *p* = 4.4 × 10^− 10^). Even more strikingly, SEPT8-X1 KD led to an almost complete absence of dendritic spines—dendrites became smooth with a few focal swellings at distal ends (Fig. 6b, f; *p* =7.0 × 10^− 82^). These abnormalities were partially rescued by co-expression of the shRNA-resistant SEPT8-X1, but not SEPT8-X5 or the palmitoylation-deficient SEPT8-X1-mut (Fig. 6c, e, f). Therefore, we conclude that SEPT8-X1 promotes dendritic branching and spine formation through its palmitoylated FIM.

Since NOVA promotes the SEPT8-X1 isoform, we predicted that neurons lacking NOVA would exhibit a similar reduction in dendrite arbor and spine density. We knocked down NOVA2 using an shRNA (shNova2) which efficiently depleted 76% of the endogenous NOVA2 in N2A cells (Additional file 2: Figure S7D). Similar to SEPT8-X1 KD, neurons depleted of NOVA2 displayed greatly decreased dendritic arbors compared to control shRNA transfected neurons, as evidenced by a 55% reduction in the number of dendritic branching points, a 47% reduction in total dendritic length, and a 48% reduction in the sholl analysis critical value (Fig. 6b, e; *p* = 3.6 × 10^− 7^, 2.5 × 10^− 7^, 1.8 × 10^− 7^, respectively). On the other hand, to our surprise, NOVA2 knockdown did not significantly affect dendritic spine density (Fig. 6b, f). Co-expression of SEPT8-X1, but not the two non-palmitoylated variants, partially restored dendritic arbor complexity in neurons with NOVA2 KD (Fig. 6d, e), suggesting that NOVA2 enhances dendritic arborization through promoting the FIM-containing SEPT8 isoform.

## Discussion

Understanding brain function involves understanding its parts, as demonstrated by the discovery of differences in ribosome-associated transcripts by looking at specific neuronal cell types in the basal ganglia (D1 vs D2 neurons) [12]. Here we develop a general method combining BAC-transgenic engineering and CLIP that is conceptually applicable to the study of any protein–RNA interactions within specific cell types. We apply this strategy to analyze differential NOVA binding in mouse spinal MNs, compare that to interactions visible at the gross level of whole spinal cord analysis, and uncover NOVA-regulated biology specific to MNs. MN-specific CLIP revealed a major role for NOVA in regulating cytoskeleton interactions in MNs, a function obscured in previous whole tissue-based analyses. This led us to discover a consequent defect in dendritic morphology in neurons lacking NOVA, which may help explain in part the severe MN defect seen in NOVA1/2 double KO MNs [21].

### MN-specific CLIP unmasks previously undetected aspects of NOVA function

MNs are among the largest neurons in the central nervous system. Their distinct morphology, characterized by a long axon and intricate dendritic arbor, renders traditional cell purifications based on enzymatic tissue digestion and cell purification methods particularly unsatisfactory for understanding the molecular biology of the whole neuron, especially given abundant evidence for RNA localization within the dendritic arbor. The cell type-specific CLIP strategy developed here allows robust and quantitative identification of NOVA binding sites in MNs in vivo, in both the cell bodies and processes. This generated a transcriptome-wide NOVA–RNA interaction atlas in MNs with NOVA binding sites in over 4000 genes. NOVA binding in MNs reflects MN transcriptome signatures, which further demonstrated the cell type specificity of our assay.

The cell type-specific CLIP strategy developed here allowed delineation of a subset of MN NOVA targets from WSC. These MN NOVA targets are enriched in genes encoding synaptic functions to a similar extent compared to WSC NOVA targets, yet they are especially enriched in genes encoding cytoskeleton interacting proteins. Enrichment of MN versus WSC CLIP signals in this subset of genes is significant even when adjusted to MN/WSC transcript level differences (*p* < 2.2 × 10^− 16^; Additional file 2: Figure S8), suggesting that these gene ontology (GO) enriched NOVA target genes contain quantitatively strengthened NOVA binding sites in MNs compared to WSC. Furthermore, through combining cell type-specific CLIP with MN transcriptome profiling, we discovered RNA processing events differentially regulated by NOVA in MNs. Around 2% of NOVA binding sites were over- or under-represented in MNs, which is consistent with our findings that ~ 2% (eight alternatively splicing regions out of 303) of NOVA-regulated alternative splicing showed MN-specific splicing patterns correctly predicted by MN-specific NOVA binding. Interestingly, the vast majority of these events also reside in transcripts encoding cytoskeleton-interacting proteins. These findings reveal a previously undiscovered cell type-specific role for NOVA in regulating MN cell biology.

### NOVA plays an important role in regulating MN cytoskeleton interactions

The top NOVA-regulated ALE was in *Sept8*, which encodes a member of the multifunctional septin family that is capable of regulating neurite outgrowth and branching through interactions with cytoskeleton components [54–56]. NOVA binding at the polyadenylation site in *Sept8* exon 10a correlated with exon 10b usage, and this was only evident in analysis of MN CLIP, not WSC CLIP alone. Consistent with prior observations of position-dependent effects of NOVA on APA [16], we demonstrated that NOVA directly inhibits cleavage/polyadenylation by binding close to the exon 10a poly(A) site, presumably promoting exon 10b transcription and splicing. Interestingly, the NOVA-dependent exon 10b encodes an FIM capable of promoting dendritic branching and spine formation. Indeed, we discovered that SEPT8-X1, the NOVA-dependent SEPT8 isoform harboring the FIM, specifically promotes dendritic arborization and spine formation. Moreover, we found that neurons lacking NOVA2 displayed decreased dendritic arbors, which are partially rescued by SEPT8-X1. Taken together, these data indicate that NOVA promotes dendritic arborization through *Sept8* regulation.

While SEPT8-X1 promotes dendritic spine formation, we were not able to detect changes in dendritic spine density in NOVA2 KD neurons. This may be related to insufficient effects from NOVA2 KD, or from contributing indirect factors, such as NOVA promotion of protein isoforms with antagonistic effects on spine formation. For example, based on in silico palmitoylation site prediction [59], we identified a total of eight NOVA targets where a predicted FIM is regulated by NOVA. Of these eight genes, NOVA promotes the FIM-harboring isoform in four (*Sept8*, *Ccp110*, *Sdccag3*, and *Ccdc84*), while inhibiting the FIM-encoding exon in the other four (*Sgce*, *Clip1*, *Kcnma1*, and *Ube2e2*). It is possible that changes of various NOVA targets upon NOVA KD mitigate the overall effect on dendritic spine density.

We have previously shown that NOVA proteins play an essential role in MN physiology [21]. MNs in mice lacking both *Nova* family members were paralyzed and failed to cluster acetyl-choline receptors at the neuromuscular junctions [21]. Successful rescue of the neuromuscular junction defect was evident after *Nova*-knockout MNs were engineered to constitutively express the Nova-regulated Z+ alternatively spliced isoform of agrin, but surprisingly remained paralyzed. Phrenic nerve stimulation revealed that the axonal synaptic machinery for conducting action potentials and synaptic vesicle release from the axon was functional, leading to the conclusion that dysregulation of additional MN NOVA RNA targets contribute to a proximal physiologic defect in *Nova*-KO MNs. Here, MN-specific CLIP reveals a major role of NOVA in regulating the MN cytoskeleton, including promoting dendritic complexity. Dendrites are the main information-receiving sites of neurons. Spinal MNs, as the gateway controllers of the CNS motor outputs, have elaborate dendritic structures to meet the highly complex demand of precisely coordinating muscle contractions spatially and temporally [63, 64]. Early and progressive dendritic degeneration has been reported in lower MNs in MN disease mouse models as well as amyotrophic lateral sclerosis (ALS) patients [65, 66], suggesting the importance of dendritic integrity to MN function. Our new findings may provide additional avenues for understanding the role that NOVA and RNA regulation plays in MN function.

### Differential NOVA binding sites in MN suggests combinatorial control of multiple RNA-binding proteins

When we compared the NOVA–RNA interactions in MNs and WSC, we uncovered over 2000 sites transcriptome-wide that are differentially bound by NOVA in MNs compared to WSC. In this way cell-specific CLIP offers the possibility of revealing cell type-specific regulatory phenomena that are otherwise obscured from analysis of whole tissues. What underlies this unique NOVA binding profile in MNs? It has been shown that combinatorial control is integral in RNA processing regulation. Cellular RNA-binding protein networks play a pivotal role in determining target selection and binding dynamics of a given RNA-binding protein [67]. For NOVA, interactions with PTBP2, the neuronal polypyrimidine tract binding protein, as well as RBFOX proteins have previously been demonstrated [20, 68]. In particular, we have shown that PTBP2 interacts with and antagonizes NOVA in the regulation of glycine receptor alpha 2 subunit (*Glra2*) splicing [68]. Interestingly, compared to all NOVA binding sites, sequences around NOVA bindings sites that are over-represented in MNs showed enrichment of pyrimidine-rich motifs and higher evolutionary conservation (Fig. 3e, f). This sequence signature around MN-specific NOVA binding sites along with lower PTBP2 levels in MNs [36] suggests the hypothesis that lower abundance of PTBP2 in MNs allows for unmasking of certain NOVA binding sites that are otherwise occupied by PTBP2 in other neurons. This kind of intricate cell type-specific interaction network may help determine *Nova* target selection beyond the specificity determined by sequence and structural constraints.

### Cell type-specific CLIP

Cell type-specific CLIP is in general applicable to any cell type and a great variety of RNA-binding proteins. The current study is neuron-specific due to the nature of *Nova* [25, 69], but a further array of cell types within the CNS or other tissues can be studied in vivo through epitope tagging. Indeed, Schaefer and colleagues used a similar strategy to identify AGO-bound miRNAs in D2 neurons of the mouse striatum [70], and *Camk2a*-neurons in the forebrain [71]. And as CLIP is applied to identify functional protein–RNA interactions defining the actions of an increasing number of RNA-binding proteins, including splicing factors [19], RNA-editing factors [72], and epigenetic regulators [73], expanding aspects of post-transcriptional and RNA-related regulation can be investigated in a cell type-specific manner. We recently described a method, PAPERCLIP, in which PABPC1 CLIP was used to identify brain-specific transcripts and alternative 3′ UTR processing within those cells [74]. Combining a cell-specific tagging strategy with PAPERCLIP would allow profiling of cell-specific polyadenylated transcripts in the brain or within any tissue, an approach that would complement the delineation of ribosome-associated transcripts demarcated by BAC-TRAP.

We examined whether GFP-NOVA2 expression in our BAC-transgenic MNs could have affected our observations relative to unperturbed neurons. The BAC-transgenic system has intrinsic expression biases and might, for example, have skewed the MN NOVA-PTBP ratios, impacting the observed NOVA binding increases around pyrimidine-rich regions. However, the high concordance between NOVA binding changes in our transgenic MNs and differential splicing in MNs without exogenous NOVA strongly argues for the physiological relevance of our transgenic model. A more elegant approach of studying cell type-specific RNA–protein interactions would be to generate conditionally epitope-tagged knock-in lines followed by the introduction of a cell type-specific Cre recombinase expression. Epitope-tagged protein would be expressed in the desired cell type from its endogenous promoter, thus preserving protein stoichiometry; this strategy has already shown promise with the ubiquitous RNA-binding protein PABPC1 [75].

Caution should be taken when comparing cell type-specific RNA binding profiles across different cell types or tissues. One issue is that differences in CLIP library complexities between comparison groups may lead to unequal statistical power in identifying over- versus under-represented binding sites. In our case, although we pooled spinal cords from multiple transgenic mice per biological replicate and performed twice as many biological replicates as the WSC CLIP, we were only able to generate less than one-eighth of CLIP reads from MN-specific CLIP compared to WSC CLIP. This is likely due to low abundance of MN-specific GFP-NOVA2 compared to the WSC NOVA pool (Fig. 1b). We hypothesize that the unevenness of library complexities contributed to the identification of a high percentage (> 72%) of NOVA binding sites significantly over-represented in MNs compared to WSC (Fig. 3a, b). We predict that achieving higher MN CLIP library complexity by including more BC and/or pooling more samples per BC can alleviate this bias.

Another challenge of analyzing cell type-specific CLIP data involves obtaining the matching cell type-specific transcriptomes, which is essential for teasing out functionally important protein–RNA interactions. A variety of technologies, such as fluorescence-activated cell sorting, LCM, and TRAP-seq can be explored for this purpose. We utilized a published RNA-seq dataset on laser captured MNs for the MN-specific transcriptome. Our transcriptome reference for WSC NOVA CLIP, on the other hand, is less than ideal. Since NOVA expression is restricted in neurons, the ideal dataset would be neuron-specific transcriptome profiling generated using technologies such as TRAP-seq or cTag PAPERCLIP.

Cell type-specific CLIP may be relevant for the study of many neurological diseases, such as ALS and ataxias, where defined cell types are pathologically affected during the entire course or the initial stages of disease progression. Interplay between the affected cells and their cellular context plays an important role in disease progression [76–79]. Our strategy allows for in vivo dissection of both cell autonomous and non-autonomous effects resulting from disease-causing mutations or insults, which had not been possible before. Great insights on cell type contributions to disease pathogenesis will be gained upon application of this strategy to a variety of animal models.

## Conclusions

Here we demonstrate the feasibility and physiological relevance of delineating neuronal cell type-specific RNA regulation. Through cell type-specific epitope tagging of the RNA-binding protein NOVA2, we generated a MN-specific NOVA–RNA interaction map using CLIP. Cell type-specific CLIP revealed a major role of NOVA in regulating cytoskeleton interactions in MNs, including promoting the palmitoylated isoform of a cytoskeleton protein, Septin 8, which enhances dendritic arbor complexity. MN-specific NOVA binding predicts MN-specific alternative RNA processing, further supporting the idea that cell type-specific RNA regulation contributes to cell identity. Our study highlights a non-incremental gain of knowledge moving from whole tissue- to single cell type-based RNA regulation analysis. As our strategy for cell type-specific CLIP is highly adaptable, we envision wide application of this method in a variety CNS cell types as well as disease models.

## Methods

### Antibodies and dilutions

The following antibodies were used in CLIP experiments: anti-GFP mouse monoclonal antibodies 19F7 and 19C8 (Memorial Sloan Kettering Monoclonal Antibody Facility), anti-NOVA serum from a paraneoplastic opsoclonus-myoclonus ataxia (POMA) patient. The following antibodies and dilutions were used for immunoblotting: anti-NOVA patient serum (1:2000), mouse anti-GAPDH monoclonal antibody 6C5 (Abcam, 1:20,000), rabbit anti-RBFOX3 (1:500) [80], rabbit anti-HA monoclonal antibody C29F4 (Cell Signaling Technology, 1:100). The following antibodies and dilutions were used for immunofluorescence: rat anti-GFP monoclonal antibody (nacalai tesque, 1:1000), goat anti-GFP polyclonal antibody (Rockland, 1:500), goat anti-CHAT polyclonal antibody (EMD Millipore, 1:500), rabbit anti-HA monoclonal antibody C29F4 (Cell Signaling Technology, 1:1000), rabbit anti-SEPT8 monoclonal antibody (pan SEPT8, EPR16099, 1:200), mouse anti-SEPT8_X1 monoclonal antibody D-11 (Santa Cruz, 1:50), chicken anti-MAP2 antibody (Thermo Fisher Scientific, 1:1000).

### Cell culture and transfection

NIH3T3 and COS-1 cells were cultured in Dulbecco’s modified Eagle’s medium (DMEM) supplemented with 10% heat-inactivated fetal bovine serum, 100 U/mL penicillin, and 100 μg/mL streptomycin. NIH3T3 and COS-1 cells were transfected with plasmid constructs using lipofectamine 2000 (Invitrogen), according to the manufacturer’s instructions.

Mouse hippocampal neurons were isolated from 18-day-old CD1 mouse embryos and cultured in 24-well plates according to established protocols. For shRNA knockdown and rescue experiments, 0.4 μg of shRNA vector and 0.6 μg of protein expressing vector or control were transfected at DIV 10 using 3 μL of NeuroMag following the manufacturer’s protocol. Transfected neurons were fixed at DIV 12 for immunofluorescence staining and confocal imaging (see below).

### Construction of BAC-transgenic mouse lines expressing GFP-NOVA2 in MNs

In brief, AcGFP-fused *Nova2* coding sequence was cloned into a pLD53.SC2 plasmid. Subsequently, a DNA fragment homologous to *Chat* 5′ UTR region was PCR amplified using primers GCCAGGCATCTGAGAGGC and CCTAGCGATTCTTAATCCAGAGTAGCAGAGCTG and inserted into the plasmid through blunt end ligation at AgeI site. The sequence of the resulting plasmid pLD53.SC2-AcGFP-Nova2 was confirmed by Sanger sequencing and deposited to the Addgene database. Recombinant BAC was generated using RP23-246B12 and pSC2-GFP-Nova2 as described previously [81] and microinjected into pronuclear oocytes of C57BL/6 mice from Charles River. By PCR genotyping, nine mice from 68 offspring were confirmed to carry the transgene. Two founders, #6 and #17, were bred with C57BL/6 mice from Charles River to establish stable transgenic lines.

### Immunofluorescence staining

Paraformaldehyde-fixed spinal cords were transversely sectioned at 14 μm thickness and kept at − 80 °C. Before immunofluorescence staining, sections were rehydrated in PBS for 10 min, followed by a 1-h block in blocking buffer containing 100 mM Tris-HCl, pH 7.5, 150 mM NaCl, 0.2% Triton X-100, 5% horse serum. Primary antibodies diluted in blocking buffer were subsequently added to the sections followed by overnight incubation at room temperature. Secondary antibody hybridization and TO-PRO-3 staining were performed using Alexa Fluor conjugated antibodies diluted in 100 mM Tris-HCl, pH 7.5, 150 mM NaCl, 0.2% Triton X-100 with 1 μM TO-PRO-3. Confocal images were taken using an inverted LSM 510 laser scanning confocal microscope (Zeiss).

For immunofluorescence staining on hippocampal neurons, cells were fixed in 4% paraformaldehyde at room temperature for 10 minutes, permeabilized with 0.1% Triton-X 100 in PBS at room temperature for 15 minutes, and blocked in PBS containing 0.1% Tween-20 and 10% donkey serum for 1 h at room temperature. The primary antibodies, diluted in PBS containing 1% BSA and 0.1% Tween-20, were added to the cells and incubated overnight at room temperature. Alexa Fluor conjugated secondary antibodies (Jackson Immunoresearch) were used at 1:500 dilution to detect either the tag epitope or proteins of interest. Confocal images were taken using an inverted LSM 880 NLO laser scanning confocal microscope (Zeiss).

### HITS-CLIP experiments and analysis

Spinal cords were hydraulically extruded in Hank’s Balanced Salt Solution (HBSS) and triturated seven times with a 21½ gauge needle in 10 mL HBSS. Immediately after trituration, tissue suspensions were crosslinked 400 mJ/cm^2^ three times at 0 °C. Crosslinked tissues were collected by centrifugation at 4000 g for 2 minutes. HITS-CLIP experiments were performed as previously described [16, 26] with PCR primers specified in Additional file 7. For MN-specific CLIP, 400 μL of Dynabeads protein G (Invitrogen) precoated with 50 μg of each anti-GFP monoclonal antibody (mAb) was used to immunoprecipitate GFP-NOVA2 in UV crosslinked spinal cord lysate pooled from five to seven 3-month-old BAC-transgenic mice. For WSC CLIP, 400 μL of Dynabeads protein G precoated with 80 μL of human anti-NOVA serum was used to immunoprecipitate lysate from one 3-month-old wild-type mouse spinal cord. cDNA libraries were prepared as previously described and sequenced on an Illumina Genome Analyzer IIx, HiSeq 1000 or HiSeq 2000.

Bioinformatic processing and mapping of CLIP NGS reads were performed similarly as previously described with slight modifications [18, 80, 82, 83]. Specifically, we removed the 3′ linker sequence from CLIP reads before mapping the remaining sequences to the mm9 build of the mouse genome with novoalign (http://www.novocraft.com) without iterative trimming using parameters –t 85 -l 25 -s 0 -o Native -r None. Following mapping, pentamer frequencies immediately downstream of CLIP reads were ranked. CLIP reads immediately upstream of the top 20 pentamers with zero or one mismatch to GTGTC were removed. We have recently discovered that under our reverse transcription (RT) conditions, the 3′ end of the RT primer could prime reverse transcription by hybridizing to GUGUC or similar pentamers in the CLIPed RNA, leading to their preferential amplification (Park C., personal communication). Bioinformatically removing such reads circumvents this technical caveat.

NOVA binding peaks were defined as previously described using gene regions compiled from refseq, UCSC known genes, and ESTs plus their downstream 10 kb as transcription units [26, 27, 80]. Gene regions with Entrez IDs were referred to as known genes. Defined peaks were then filtered for biological complexity, requiring CLIP reads from at least half of the biological replicates. Joint peaks (JPs), in particular, were defined by running the peak finding algorithm using CLIP reads pooled from both comparison groups (e.g., MNs and WSC), and requiring CLIP reads from at least half of the biological replicates in either group (i.e., WSC BC 2 out of 4 or MN BC 4 out of 8).

Gene-wise CLIP read enrichment between WSC and MNs was evaluated using the Bioconductor edgeR package with tagwise dispersion model [29]. *P* values of differential NOVA binding on specific sites were calculated using Fisher’s exact test on the following 2 × 2 matrix:

$$ {\displaystyle \begin{array}{cc}\mathrm{m}& \mathrm{M}\hbox{-} \mathrm{m}\\ {}\mathrm{n}& \mathrm{N}\hbox{-} \mathrm{n}\end{array}} $$

where m and n stand for the number of MN and WSC CLIP reads in a given JP, respectively, while M and N stand for the total number of intronic/exonic MN and WSC CLIP reads within all intronic/exonic JPs in the corresponding transcript, respectively. Coverage is defined as the smallest number among the 2 × 2 matrix. Benjamini-Hochberg multiple-testing correction was performed using all intronic or exonic JPs with a minimum coverage of 10.

### RNA-seq library preparation and data analysis

We performed WSC RNA-seq on two biological replicates of 3-month-old SOD1^WT^ transgenic mice in SJL/B6 mixed background. For each replicate, poly(A) selected RNA from spinal cord was used to prepare RNA-seq library using an Illumina TruSeq RNA sample prep kit. We generated 100-nt paired-end reads using an Illumina Genome Analyzer II. In order to compare WSC and MN RNA-seq, we trimmed our WSC reads to 74 nt to match the read length of the MN RNA-seq dataset by Bandyopadhyay et al. (GSE38820). Both WSC and MN datasets were mapped to the mouse (mm9) genome and exon junctions using OLego with default parameters (15-nt seed with 1 nt overlapping, four or fewer mismatches per read) [84]. Only reads unambiguously mapped to the genome or exon junctions were retained for downstream analysis. For MN RNA-seq, 36,484,398 and 38,293,208 NGS reads were mapped for the two biological replicates, respectively, while 74,444,847 and 81,175,598 NGS reads were mapped for WSC RNA-seq replicates. Transcript level and alternative splicing analyses were performed as previously described. Bioconductor edgeR package was used to evaluate statistical significance of transcript level differences between WSC and MNs [29]. Fisher’s exact test was used to calculate *p* values of differential alternative splicing, and the FDR was estimated using the Benjamini-Hochberg method. Alternative exon splicing was analyzed as described [26]. Alternative exons with splice junction read coverage over 10 [26] were considered “expressed”, and were included in Benjamini-Hochberg multiple test correction. Differential alternative splicing events were identified by requiring FDR ≤ 0.1 in addition to biological consistency (BC2 out of 2).

### Gene ontology analysis

GO analysis was performed using GOrilla by running unranked lists of target and background genes [85]. For background genes, we used all genes with rpkm ≥ 1 in WSC or MNs.

### Motif enrichment analysis

For motif enrichment around differential MN intronic NOVA peaks, tetramer occurrences 100 nt around the center of each peak were counted and compared to those 100 nt around all intronic or exonic NOVA peaks using a hypergeometric test.

### Sept8 minigene assay

To construct the *Sept8* minigene, the C57BL/6 genomic region amplified with primers TACGACTCACTATAGGGCGAATTCGGATCCGCATGAATTCTGACCCCTGTGA and CAATAAACAAGTTCTGCTTTAATAAGATCTCCGTAACCTGGCTACCAGTGA was cloned into BamHI and BglII digested pSG5 vector using HIFI DNA assembly kit (New England Biolabs). Mutant minigenes were constructed by assembling the PCR amplified vector backbone (forward, TTGGGCAGTAGCTTCGCTG; reverse, TAACATAACAGAGAAGCAAGCTGGCT) with synthetic gBlocks (IDT DNA) carrying the desired mutations. Minigene (1 μg) was co-transfected into COS-1 cells with 1 μg of control vector or NOVA/RBFOX3 expression vector. Cells were harvested 48 h post-transfection for immunoblotting or RT-qPCR following standard protocols. Primers CTGACCCCGCATATGTTCCTGTGT and CAAGCTGGCTATCCTGGGCCTCTT were used to amplify an exon 10a region, while primers GTCTGCGATGGTTTTGCAGAGGTG and GGCCACAGGAAATGGAGATGTGAG were used for an intron 10 amplicon. Three independent experiments were performed for statistical comparison.

### Acyl-RAC assay

We seeded 250,000 COS-1 cells per well in six-well plates and transfected them with 2 μg of constructs expressing FLAG-HA-tagged SEPT8 variants 18–24 h later. Acyl-RAC assay was performed using the CAPUREome S-Palmitoylated Protein Kit (Badrilla) according to the manufacturer’s protocol. Treated protein lysates were subsequently immunoblotted with rabbit anti-HA antibody.

### Image analysis

Maximum projected confocal images were produced using ImageJ using only linear adjustments. Dendrites were semi-automatically traced using the Simple Neurite Tracer plugin. Critical value and number of dendritic branching points were deduced by using the “sholl analysis” and “analyze skeleton” plugins. Fifteen to 17 neurons were analyzed per group.

## Additional files

Additional file 1: Supplemental material and methods. (DOCX 115 kb)

Additional file 2: **Figure S1.** GFP-Nova2 retains Nova2 RNA binding preference. **Figure S2.** HITS-CLIP on BAC transgenic spinal cords and WSC. **Figure S3** Data analysis method. **Figure S4.** Bioinformatic analyses of disproportionally different NOVA binding sites in MNs. **Figure S5.** Additional cases where MN-specific NOVA binding correlates with MN-specific alternative splicing. **Figure S6.** NOVA regulates Sept8 ALE usage. **Figure S7.** Illustration of palmitoylation detection assay; SEPT8 expression in hippocampal cultures; SEPT8-X1 and NOVA2 knockdown in cell lines. **Figure S8.** NOVA binding on genes encoding cytoskeleton interacting proteins are over-represented in MNs. (PDF 30346 kb)

Additional file 3: **a** Sequencing and mapping statistics of MN and WSC NOVA CLIP. **b** Sequencing and mapping statistics of MN and WSC NOVA CLIP. **c** mm9 coordinates and genomic annotation of WSC NOVA CLIP peaks. **d** List of known NOVA targets harboring WSC NOVA CLIP peaks. **e** List of known NOVA targets harboring MN NOVA CLIP peaks. **f** GO enrichment of known MN versus WSC NOVA targets. **g** Transcripts with enriched NOVA CLIP signals in MNs compared to WSC. **h** Transcripts with depleted NOVA CLIP signals in MNs compared to WSC. (XLSX 13044 kb)

Additional file 4: **a** Differential analysis results for all intronic joint peaks. **b** Differential analysis results for all intronic joint peaks. (XLSX 14058 kb)

Additional file 5: **a** Transcript regions differentially spliced between MNs and WSC. **b** For the nine exons with over- or under-represented MN NOVA binding sites in regulatory “hotspots”, correlation between observed and predicted dI between MNs and WSC. (XLSX 365 kb)

Additional file 6: **a** List of alternative last exons differentially utilized in MNs vs WSC. **b** List of alternative last exons differentially utilized in wild-type vs Nova2 knockout mouse brain. (XLSX 69 kb)

Additional file 7: List of RNA linkers and DNA primers used in CLIP. (XLSX 70 kb)

Additional file 8: Review history for this manuscript. (DOCX 74 kb)
