# Supplementary material for: Cell type-specific CLIP reveals that NOVA regulates cytoskeleton interactions in motoneurons
Source: Genome Biol. 2018 Aug 15;19:117. doi: 10.1186/s13059-018-1493-2 (PMC6092797; doi:10.1186/s13059-018-1493-2)
Supplement: Supplementary file 1 — Supplemental material and methods. (DOCX 115 kb) [file 13059_2018_1493_MOESM1_ESM.docx]

**Supplemental Material and Methods**

## Vector construction

AcGFP-Nova2 was subcloned from pLD53.SC2-AcGFP-Nova2 using BamHI and EcoV sites and inserted into pcDNA3.1(+) to construct pcDNA3-AcGFP-Nova2.

For pcDNA3-Nova2, Nova2 was PCR amplified from pLD53.SC2-AcGFP-Nova2 using primers ACTTAAGCTTGGTACCGAGCTCGGATCCGCCACCATGGAGCCC

GAGGCCCCG and CTGTGCTGGATATCTGCAGAATTCTCATCCCACTTTCTGTG

GGTTTGAAGC. PCR amplified fragment was assembled into BamHI and EcoRI digested pcDNA3.1(+) using NEBuilder HiFi DNA assembly kit following manufacturer’s instructions.

Sept8-X1 and X5 coding sequences were PCR amplified from C57BL6/J mouse spinal cord cDNA library using primers CGCCTCGAGATGGCGGCCACCGACTTGGAACG and CAGGCGGCCGCTCAGAGGAATCCTTCCCTCCACGTCGC (reverse for X1) or CAGGCGGCCGCTCAAGGAATAGTGACACTGTAAATGGAAGACCAGCCACC (reverse for X5), prior to cloning into pOZ-FH-N vector at the XhoI and NotI sites. Subsequently, the FLAG-HA tagged Sept8 cDNA fragments were subcloned into pcDNA3.1(+) between the HindIII and NotI sites. C469C470 – SS mutations, as well as shRNA resistant constructs were generated by assembling SfiI and NotI digested pcDNA3-SEPT8 fragments with synthetic DNA fragments (IDT DNA) carrying the desired mutations.

shRNA constructs were generated by annealing the following oligos before cloning into pSuper.gfp/neo using the BglII and HindIII sites:

scramble:

Forward:

GATCCCCTAAGGCTATGAAGAGATACTTCAAGAGAGTATCTCTTCATAGCCTTATTTTTA

Reverse:

AGCTTAAAAATAAGGCTATGAAGAGATACTCTCTTGAAGTATCTCTTCATAGCCTTAGGG

shX1:

Forward: GATCCCCGTGATGATGACTAAAGCAATTCAAGAGATTGCTTTAGTCATCATCACTTTTTA

Reverse:

AGCTTAAAAAGTGATGATGACTAAAGCAATCTCTTGAATTGCTTTAGTCATCATCACGGG

shNova2:

Forward: GATCCCCGCAAGCCGCTCAATACCTCATCAGCCATTCAAGAGATGGCTGATGAGGTATTGAGCGGCTTGCTTTTTA

Reverse:

AGCTTAAAAAGCAAGCCGCTCAATACCTCATCAGCCATCTCTTGAATGGCTGATGAGGTATTGAGCGGCTTGCGGG

## NIH/3T3 transfection and HITS-CLIP

Each 10 cm plate of NIH/3T3 cells was transfected with 10 ug of pcDNA3-AcGFP-Nova2 or pcDNA3-Nova2 plasmid using lipofectamine 3000 according to manufacture’s protocol. 48 hours post-transfection, each plate was UV-crosslinked on ice at 400 mJ/cm^2^ once. 400 uL of Dynabeads protein G precoated with 50 ug of each anti-GFP mAb or 80 uL human anti-Nova serum was used for GFP-Nova2 and untagged Nova2 IP from each plate, respectively. Two replicates were performed. cDNA libraries were prepared as previously described and sequenced on Illumina MiSeq and HiSeq 2500.
