# Supplementary material for: Cell type-specific CLIP reveals that NOVA regulates cytoskeleton interactions in motoneurons
Source: Genome Biol. 2018 Aug 15;19:117. doi: 10.1186/s13059-018-1493-2 (PMC6092797; doi:10.1186/s13059-018-1493-2)
Supplement: Supplementary file 2 — Figure S1. GFP-Nova2 retains Nova2 RNA binding preference. Figure S2. HITS-CLIP on BAC transgenic spinal cords and WSC. Figure S3 Data analysis method. Figure S4. Bioinformatic analyses of disproportionally different NOVA binding sites in MNs. Figure S5. Additional cases where MN-specific NOVA binding correlates with MN-specific alternative splicing. Figure S6. NOVA regulates Sept8 ALE usage. Figure S7. Illustration of palmitoylation detection assay; SEPT8 expression in hippocampal cultures; SEPT8-X1 and NOVA2 knockdown in cell lines. Figure S8. NOVA binding on genes encoding cytoskeleton interacting proteins are over-represented in MNs. (PDF 30346 kb) [file 13059_2018_1493_MOESM2_ESM.pdf]

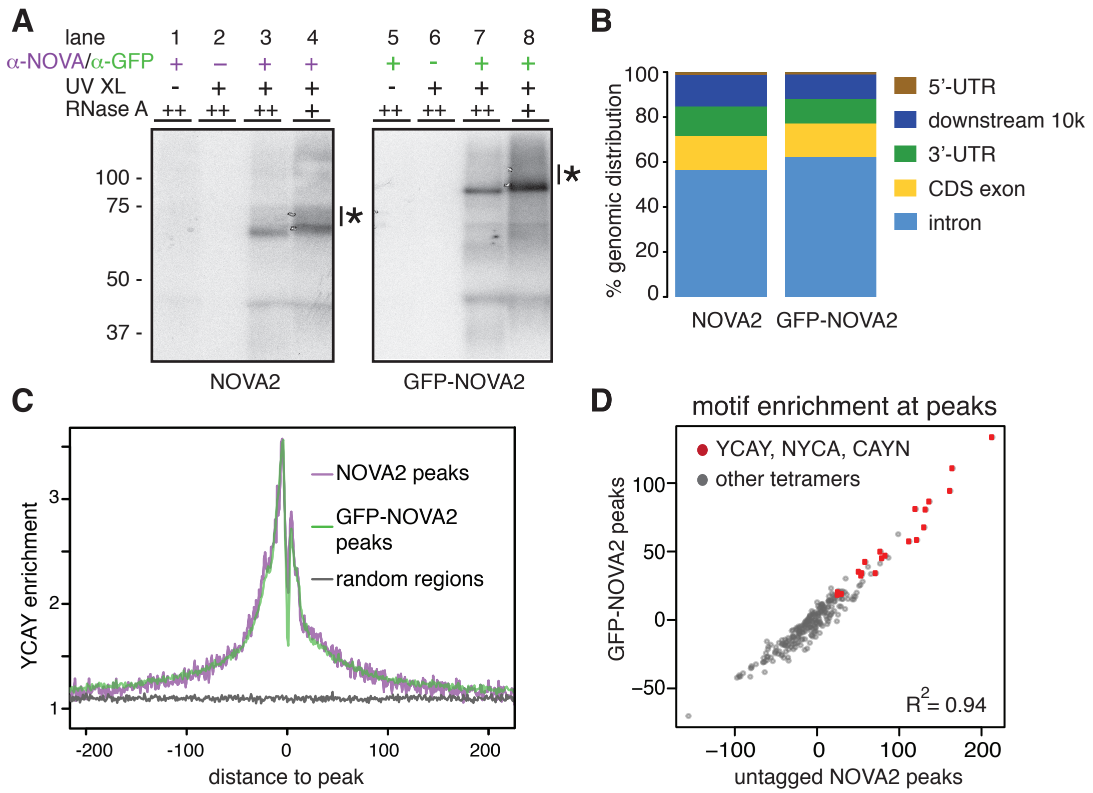

Figure S1. GFP-Nova2 retains Nova2 RNA binding preference

A. Autoradiogram showing NuPAGE separation of radio-labeled NOVA2- and GFP-NOVA2-RNA complexes immunoprecipitated from transfected NIH/3T3 cells. Both UV irradiation and use of cognate antibodies in immunoprecipitation were required for the presence of radio-labeled NOVA2- and GFP-NOVA2-RNA complexes (lanes 1-2 & 5-6), which appeared as a smear with partial RNase digestion (lanes 4 & 8) and collapsed to tighter bands upon more complete RNase digestion (lanes 3 & 7). The stars and lines mark the sizes of NOVA2- and GFP-NOVA2 -RNA complexes isolated for CLIP library preparation.

B. Genomic distribution of NOVA2 and GFP-NOVA2 HITS-CLIP reads.

C. Enrichment of YCAY motif around NOVA2 and GFP-NOVA2 CLIP peaks. YCAY enrichment is calculated by normalizing the number of YCAY starting from each position relative to CLIP peaks to the expected YCAY frequency based on random base distribution. Random regions are size matched genomic regions 10 kb away from pooled Nova2 and GFP-NOVA2 CLIP peaks.

D. Pairwise correlation of motif enrichment z-scores at NOVA2 and GFP-NOVA2 peaks. Tetramer occurrences 20 nt around NOVA2 or GFP-NOVA2 CLIP peaks were counted and z-scores were calculated using tetramer frequencies around size matched random genomic regions as background. Tetramers that may overlap YCAY by at least three nucleotides are colored red. Correlation of motif enrichment z-scores around NOVA2 and GFP-NOVA2 CLIP peaks is indicate at the lower right corner.

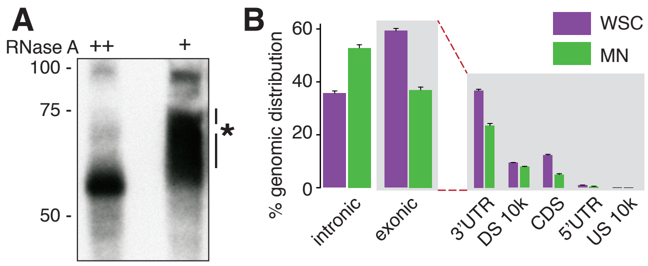

Figure S2. HITS-CLIP on BAC transgenic spinal cords and WSC.

A. Autoradiogram showing NuPAGE separation of radio-labeled NOVA-RNA complexes immuno-precipitated WSC using a human anti-NOVA serum. NOVA-RNA complexes appeared as a smear with partial RNase digestion (1st lane), and collapsed to a tighter band upon more complete RNase digestion (2nd lane). Lines mark the sizes of NOVA-RNA complexes isolated for CLIP library preparation.

B. Genomic distribution of WSC and MN HITS-CLIP peaks.

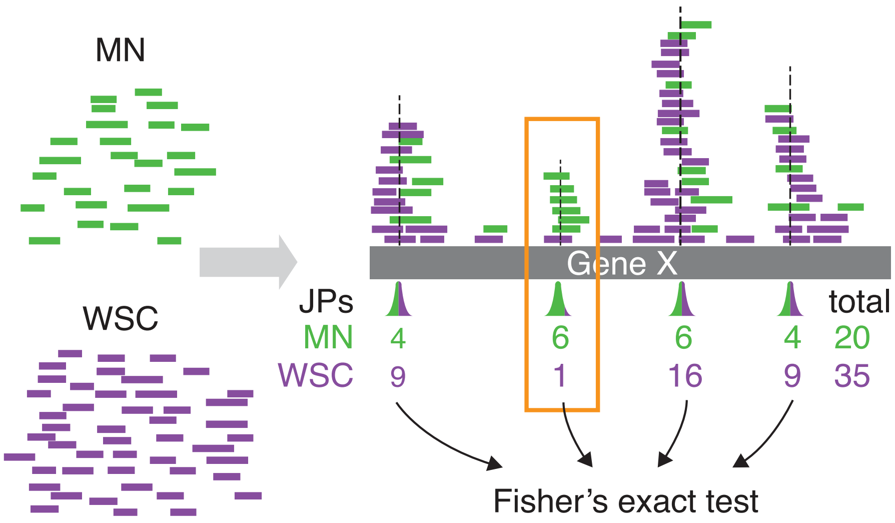

Figure S3. Illustration of data analysis method. CLIP reads from MN (green) and WSC (purple) were pooled to defined JPs, which are represented in green and purple dual-colors. For each gene with multiple JPs, CLIP reads contributions from the WSC or MN CLIP data were counted separately for each JP, as well as for the entire gene. Pairewise Fisher's exact test was performed to identify disproportionally different JPs between WSC and MN, such as the one highlighted in the orange box.

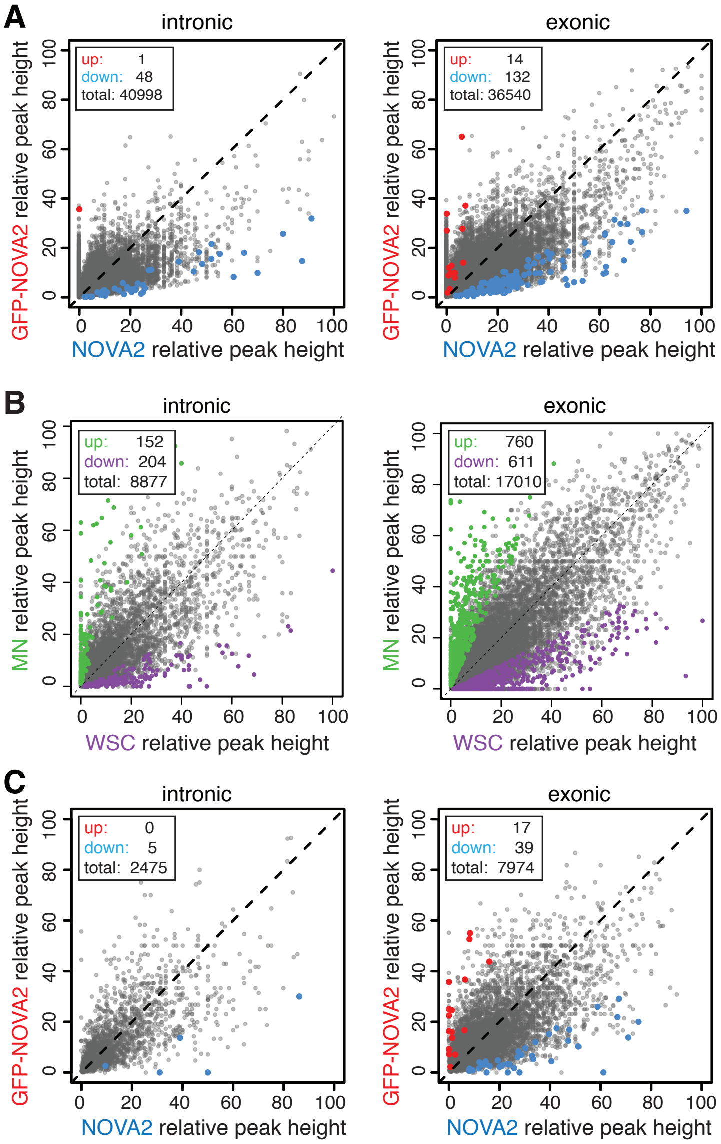

Figure S4. Bioinformatic analyses of disproportionally different NOVA binding sites in MN.

A. Pairwise comparison of intronic and exonic relative peak heights in NOVA2 and GFP-NOVA2 CLIP. Relative peak heights in introns or exons are calculated as following: 100 * number of NOVA2 or GFP-NOVA2 reads in a JP / number of NOVA2 or GFP-NOVA2 CLIP reads in all intronic or exonic JPs in the corresponding gene. Peaks differentially strengthened or weakened in GFP-NOVA2 compared to NOVA2 CLIP (FDR ≤ 0.1, fold change ≥2) are shown in red and blue, respectively. The numbers of changed peaks and total peaks with sufficient coverage for the analysis are shown in the boxes.

B-C. Pairwise comparison of intronic and exonic relative NOVA peak heights in downsampled GFP-NOVA2 and NOVA2 CLIP(B), and MN and WSC CLIP (C). MN, WSC and GFP-NOVA2 CLIP datasets were randomly downsampled to match the complexity of untagged NOVA2 CLIP, prior to analysis using the same pipeline. See Figure S4A for legend.

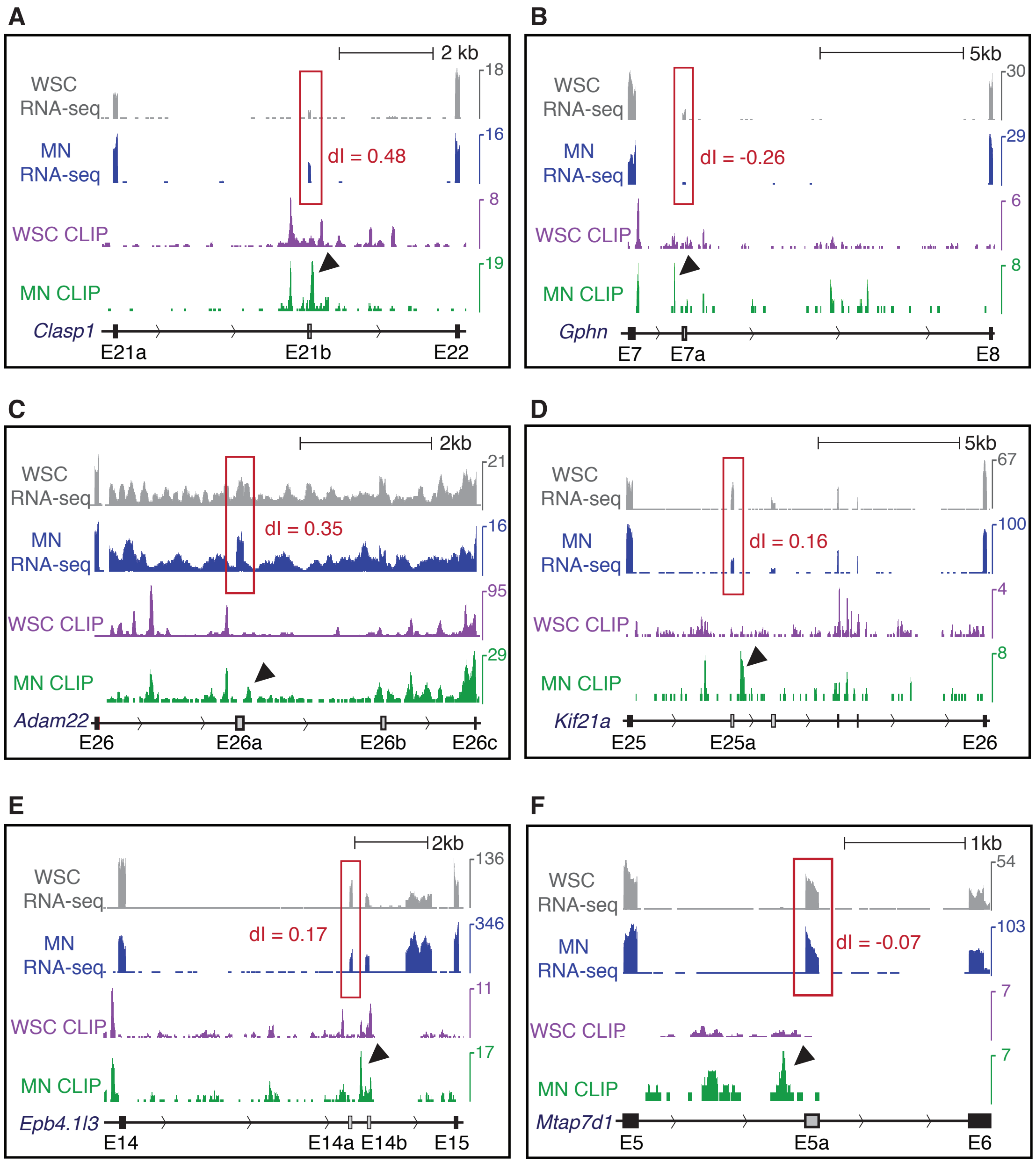

Figure S5. Additional cases where MN-specific NOVA binding correlates with MN-specific alternative splicing. UCSC genome browser images illustrating correlation between differential NOVA binding and MN specific alternative splicing in six additional genes. See Figure 4 C-D for legend.

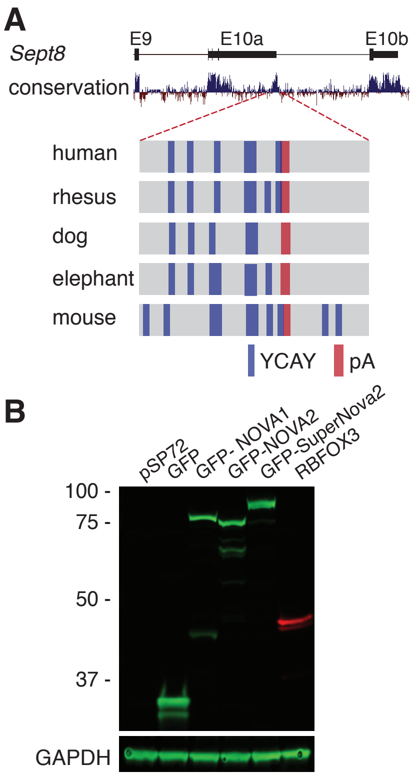

Figure S6. NOVA regulates Sept8 ALE usage.

A. The genomic region around Sept8 E10a poly(A) site is highly conserved, as shown by the Placental mammal basewise conservation by PhyloP track displayed beneath the partial Sept8 gene structure. Positions of YCAY motifs 150 nt around poly(A) signals are shown at the bottom for multiple mammals. Navy bars represent YCAY motifs, and red bars represent poly(A) sites.

B. A representative immunoblot image showing exogenous protein expressions in the Sept8 minigene assay. COS-1 cells were co-transfected with Sept8 minigene variants and protein expression vectors in duplicate plates. 48 hours post-transfection, one set of transfected cells was used for protein lysate preparation and subsequent western blot analysis using antibodies against GFP, RBFOX3 and GAPDH.

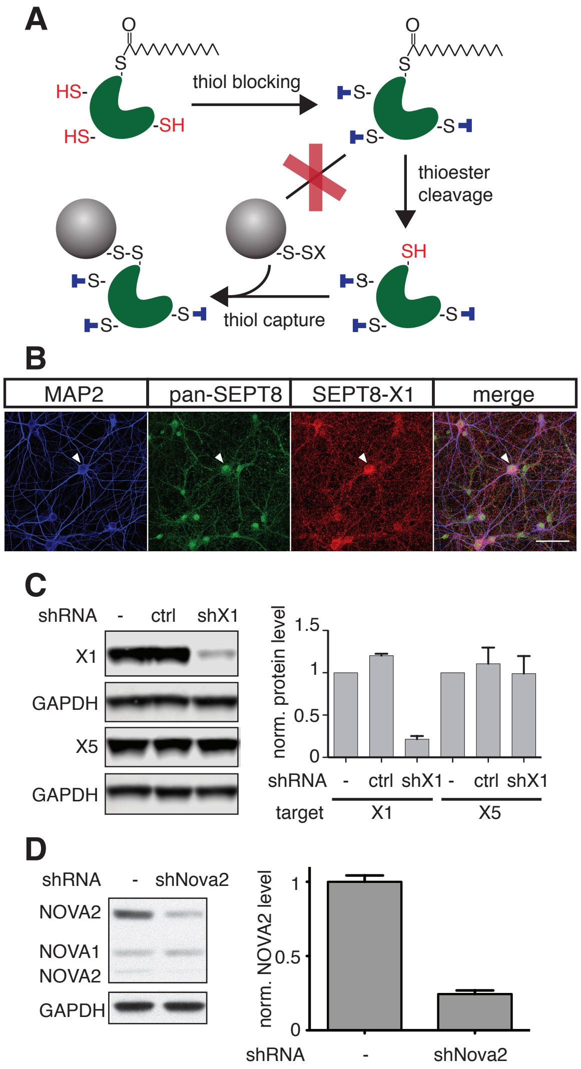

Figure S7.

A. Schematic overview of the acyl-RAC method for palmitoylation detection. Following cell lysis, free sulfhydryl groups (red) were blocked with a thiol reactive reagent (blue). Subsequently palmitate cleavage generates free sulfhydryl groups which are then captured on thiopropyl-sepharose. Samples that do not undergo palmitate cleavage following thiol blocking lack reactive sulfhydryl group, and therefore cannot be captured on thiopropyl-sepharose.

B. Representative maximum projected confocal images of DIV10 hippocampal culture, stained with antibodies against MAP2, pan-SEPT8 and SEPT8_X1 isoform. White arrowheads point to a neuron soma. Scale bar represents 50 µm.

C. Immunoblotting showing efficient knockdown of SEPT8-X1 in COS-1 cells. COS-1 cells were co-transfected with constructs expressing FLAG-HA tagged SEPT8-X1 or X5, and shRNA (empty, scramble or shX1). 48 hours post-transfection, cell lysates were used for western blot analysis using antibodies against HA and GAPDH. Normalized protein levels are shown on the right. Error bars represent SEM based on three independent experiments.

D. Immunoblotting showing efficient NOVA2 knockdown in N2a cells. N2a cells were transfected with empty or shNova2 expressing vector. 48 hours post-transfection, cells lysates were used for western blot analysis using antibodies against NOVA and GAPDH. Normalized NOVA2 levels are shown on the right. Error bars represent SEM based on three independent measurements.

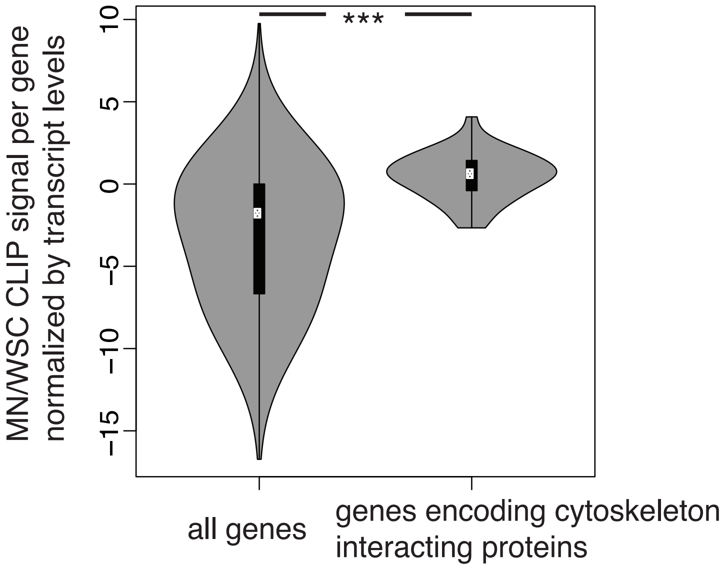

Figure S8. NOVA binding on genes encoding cytoskeleton interacting proteins are over-represented in MNs. We normalized CLIP signal enrichment between MN versus WSC by gene expression levels in MN and WSC. The resulting ratios (MN_CLIP * WSC_rpkm / MN_rpkm * WSC_CLIP) are plotted in the violin plot for all genes, or the subset of NOVA targets encoding cytoskeleton interacting proteins (p < 2.2 x 10-16, Student's t test).
