# Supplementary material for: Cell type-specific CLIP reveals that NOVA regulates cytoskeleton interactions in motoneurons
Source: Genome Biol. 2018 Aug 15;19:117. doi: 10.1186/s13059-018-1493-2 (PMC6092797; doi:10.1186/s13059-018-1493-2)
Supplement: Supplementary file 8 — Review history for this manuscript. (DOCX 74 kb) [file 13059_2018_1493_MOESM8_ESM.docx]

**First round of review**

**Reviewer 1**

**Are the methods appropriate to the aims of the study, are they well described, and are necessary controls included?**

Yes

**Are the conclusions adequately supported by the data shown?**

Yes

**Are sufficient details provided to allow replication and comparison with related analyses that may have been performed?**

Yes

**Does the work represent a significant advance over previously published studies? Please explain.**

Yes, the analysis of RNA binding events in a single cell type is a conceptual advance.

**Is the paper of broad interest to others in the field, or of outstanding interest to a broad audience of biologists?**

Yes: Will be of interest in the field of RNA biology, neuroscience, and those interested in post-translational modifications

**Comments to authors**

This manuscript by Yuan et al. represents a relatively new development by the Darnell group to extend analysis of regulatory interactions between the Nova RNA binding proteins and RNA targets at the level of a single cell type. Members of this group were pioneers in the establishment of the HITS-CLIP methods for analysis of Nova targets as well as other RNA binding proteins. While previous studies of neural RNA binding proteins were largely carried out in extracts from whole brain tissues or other neural tissues, here they use Bac-transgenic mice to express a tagged Nova2 protein specifically in spinal cord motor neurons (MNs). The extension of this technology at single cell-type resolution is a conceptual advance that will help set the stage for similar investigations of other RNA binding proteins. These studies revealed that, compared to whole spinal cord (WSC) Nova HITS-CLIP, the Nova targets in MNs were more highly enriched for transcripts that encode proteins that bind to components of the cytoskeleton. The study also uses an RNA-Seq database of MNs to compare Nova alternative splicing of Nova targets with that in whole spinal cord RNA-Seq. This analysis revealed a subset of Nova targets that are differentially splicing in MNs. Among these targets were several with patterns of Nova binding in MNs indicative of direct regulation. Finally, an analysis of alternative last exon use in MNs vs. spinal cord identified an example, Sept8, as a Nova regulated ASE that is differentially regulated in MNs. A detailed functional analysis is carried out to investigate the consequences of splicing to the more distal Sept8 ALE to produce the X1 protein isoform (which is also dependent on Nova based on previous work by the authors). This analysis shows that ALE in isoform X1 contains palmitoylation sites that are part of a filopodia inducing motif (FIM). Isoform-specific KD of this isoform in primary mouse hippocampal neurons resulted in nearly complete loss of dendritic spines as well as reduced dendritic arbors. Perhaps somewhat disappointingly, depletion of Nova in neurons resulted in decreased arbors, but no appreciable affect on dendritic spine density, thus making a link between Nova regulation of Sept8 and the phenotype from loss of the X1 isoform incomplete. While several plausible explanations are given for this, it does somewhat decrease the enthusiasm for the otherwise nice functional characterization of this alternative splicing event. Not mentioned is the possibility that this aspect of Nova regulation in MNs is not recapitulated in primary hippocampal neurons. I do not have any major criticism of the study but several small points to make that might be addressed to add some clarity for some readers.

-One of the main limitations of the type of cell type analysis carried out here is the understandably relatively small numbers of unique CLIP reads and peaks obtained from MNs. This issue bears some further discussion including alternative approaches or technical advances that might overcome this issue. For example, perhaps larger numbers of animals per replicate. This issue also bears some discussion in the analysis of peaks that are compared between MNs and WCSs.

-It is not intuitive how the analysis of Nova CLIP tags is used to verify the specificity of CLIP for either MNs or whole spinal cords. Is there a reason to expect that MN specific targets such as Chat would be directly targeted, or regulated by Nova? Or is the presence of tags (whether or not present in peaks) simply being used as a surrogate for gene expression? Similarly it is not clear given the limited depth of CLIP reads in MNs whether the absence of inhibitory neuron markers such as Slc6a5 or Erbb4 is indicative of specificity. Some added commentary might help clear this up.

-The use of the terms "strengthened" or "weakened" to refer to Nova binding sites in MNs vs. WSCs does not seem to be the best way to describe these sites since strength of binding is not being measured. It would seem use terms such as over- or under-representation might be more appropriate.

-The examples shown in Fig. 4 C and D are informative regarding the subset of MN specific events with evidence for direct Nova regulation. It would also be useful if the authors could indicate the calculated Percent Spliced I (PSI) for these exons to give a clearly picture as to the degree of difference in splicing in each dataset. It would also be useful if the same schematics could also be shown in a supplemental figure for the other 6 of these events.

-It would be helpful if the authors could describe or show the stoichiometry of Nova2 expression levels in MNs compared to endogenous Nova (beyond that in whole spinal lysates shown in Fig. 1B). Are the levels similar to endogenous, lower, overexpressed? This would be expected to impact the specificity as well as yield of the MN-specific CLIP observed.

**Reviewer 2**

**Are the methods appropriate to the aims of the study, are they well described, and are necessary controls included?**

Mostly yes; some details need clarification.

**Are the conclusions adequately supported by the data shown?**

The authors should point out alternative explanations as outlined below, and add some analyses, but overall fine.

**Are sufficient details provided to allow replication and comparison with related analyses that may have been performed?**

Broadly, yes, but see the detailed comments below.

**Does the work represent a significant advance over previously published studies?**

The manuscript describes an approach to combining CLIP with a mouse transgenic line expressing a tagged protein of interest under a cell-type specific promoter. It is the first cell-type specific RBP-RNA interaction profile, and the study demonstrates technical feasibility of performing a CLIP experiment, which is sensitive to material limitation, on an RBP expressed in small cell numbers.

**Is the paper of broad interest to others in the field, or of outstanding interest to a broad audience of biologists?**

Yes: The study will be of high interest both to the post-transcriptional regulation field and the neuroscience community, the cell type being cholinergic motoneurons of the spinal cord, involved in multiple neurodegenerative diseases.

**Comments to Author:**

Yuan et al. provide a cell-type-specific RNA-binding protein (RBP) - RNA interactome, combining a previously developed CLIP method with a mouse transgenic line expressing a tagged protein of interest under a cell-type specific promoter. It is the first cell-type specific RBP-RNA interaction profile of this kind. Importantly, the study demonstrates technical feasibility of performing a CLIP experiment, which is sensitive to material limitation, on an RBP expressed in just a subset of cells in the tissue. 

The Nova2 targetome in motoneurons will be a valuable resource for starting to understand cell type-specific RNA regulation. In addition, one single outstanding target, Septin 8, is followed up in detail, revealing a novel post-translational modification of a Nova2-dependent isoform of this protein, which has a drastic impact on neuronal morphology.

The study is conducted carefully with the necessary controls present. The data presented is of excellent quality. 

General comments:

- One should be careful about interpreting the results of alternative splicing analysis. The authors compare MN CLIP with WSC CLIP and match it to alternative splicing analysis of the MN and WSC RNA-Seq. Whereas motoneuron CLIP and MN RNA-Seq are indeed matching datasets, WSC RNA-Seq comprises RNA from all cell types of the spinal cord, neuronal and non-neuronal, whereas WSC CLIP only corresponds to the cell types that express Nova2 (presumably, only neurons). This needs to be clarified and addressed properly.

Specific comments:

- The GO enrichment in terms "microtubule binding", "tubulin binding" and "cytoskeletal protein binding" (Fig. 2E) may not be due to some of these targets being found specifically in motoneurons. Rather, the total number of Nova2 targets identified in motoneurons (MN) was less (173) than in the whole spinal cord (WSC) (301), and the depth of MN CLIP datasets is several fold less than that of the WSC CLIP datasets. Cytoskeleton-related proteins tend to be highly expressed and might have avoided under-detection relative to other targets. As such, you cannot use these datasets for direct comparisons such has GO without further normalization (eg subsampling). Do these target genes contain also quantitatively strengthened MN peaks?

- The effect of knocking down Sept8-X1 isoform on the formation of dendritic spines is striking and very specific. Therefore, it is indeed surprising that Nova2 knockdown has no measurable effect on dendritic spine numbers. The authors discuss that this could be because of either not sufficient effect of Nova2 knockdown or other targets of Nova2 possibly having antagonistic effect on spine formation. An alternative explanation could be that in hippocampal neurons, as opposed to motoneurons, Nova2 does not affect splicing of Sept8-X1 isoform, and Nova2 KD effect on arborization is through other Nova2 targets (among which are many cytoskeleton related proteins), which is partially mitigated by Sept8-X1 overexpression. Does Sept8-X1 overexpression by itself increase arborization? (for example, in scrambled shRNA control?) Also, immunostaining of Sept-X1 and Nova2 KD hippocampal neurons with X1-specific antibody could help to access the efficiency of Sept8-X1 isoform depletion in this neuronal type.

Minor comments:
- For experimental reproducibility, more details need to be included on how exactly spinal cords were processed for CLIP. Were they crosslinked as cell suspension? How was tissue dissociation performed? Was the integrity of the cells microscopically checked during tissue dissociation? Since the manuscript claims to have quantified Nova2 binding "in both the cell bodies and processes", this needs to be clarified.

- Also, please be more detailed in your computational analysis, eg peak calling. It shouldn't be necessary for the reader to consult multiple manuscripts to consolidate "previously described" approaches (I would be very much (positively) surprised if the data analysis across all these previous studies was completely identical and thus unambiguous and reproducible). 

- It is not very clear from the text that Nova2 protein apparently has two isoforms, longer and shorter (Western blot in Fig. 1B). Whereas GFP-Nova2 construct and thus motoneuron (MN) CLIP was made with the short isoform, it is not explicitly mentioned with which isoform(s) the whole spinal cord (WSC) CLIP on endogenous Nova2 was done. It appears from the radioactive gel in Fig. S2A that it was the long isoform (cf. Fig. 1B), but a clarification, and a discussion why which isoform was chosen, would be helpful.

- Gene names such as Sept8 and March1 have been automatically converted to date by Excel in supplementary tables (e.g. Additional File 1, Sup_1B).

- Vertical range scale for coverage is missing on the figures that contain genome browser screenshots (Fig. 4C,D, 5C).

(- Many relative MN peak heights as in Fig. 3A,B seem to show discrete values. That possibly might arise from joined peaks that contain low MN read numbers but are being called due to sufficient reads present in the WSC dataset. It might be beneficial to increase stringency for filtering on minimal number of reads coming from each respective sample.) 

- The discussion focuses on a phenotype of Nova1/Nova2 double knockout mouse but does not address a possible degree of mutual compensation between these RBPs. A discussion of motoneuron phenotype specifically of Nova2 knockout mouse in the context of the MN-Nova2-targetome is missing.

**Authors’ response to reviewers**

**Response to Reviewer 1:**

1. “*One of the main limitations of the type of cell type analysis carried out here is the*

*understandably relatively small numbers of unique CLIP reads and peaks obtained from*

*MNs. This issue bears some further discussion including alternative approaches or technical*

*advances that might overcome this issue. For example, perhaps larger numbers of animals*

*per replicate. This issue also bears some discussion in the analysis of peaks that are*

*compared between MNs and WCSs.”*

The reviewer has an excellent point, and we have added a paragraph in Discussion (P24-25).

*2. “It is not intuitive how the analysis of Nova CLIP tags is used to verify the specificity of*

*CLIP for either MNs or whole spinal cords. Is there a reason to expect that MN specific*

*targets such as Chat would be directly targeted, or regulated by Nova? Or is the presence of*

*tags (whether or not present in peaks) simply being used as a surrogate for gene expression?*

*Similarly it is not clear given the limited depth of CLIP reads in MNs whether the absence of*

*inhibitory neuron markers such as Slc6a5 or Erbb4 is indicative of specificity. Some added*

*commentary might help clear this up.”*

The presence of at least some statistically significant binding of NOVA to spinal cord

transcripts is seen by calling NOVA binding sites within 12,445 genes in WSC CLIP, and the

distribution of Joint Peaks among 13,559 genes. This is consistent with our observation of

one or more robust NOVA binding sites on over half of expressed transcripts. Perhaps more

importantly, and to the Reviewer’s point, since we find that transcript abundance positively

correlates with CLIP signal strengths, we used the total number of individual tags within

NOVA binding sites per transcript as a rough surrogate for relative levels of gene expression.

This is now clarified in P8, lines 13-14. The depletion of NOVA CLIP reads on inhibitory

neuron markers is highly statistically significant, which was calculated using edgeR under

the negative binomial model, taking into account different read depths of both MN and WSC

CLIP.

*3. “The use of the terms "strengthened" or "weakened" to refer to Nova binding sites in MNs vs.*

*WSCs does not seem to be the best way to describe these sites since strength of binding is not*

*being measured. It would seem use terms such as over- or under-representation might be*

*more appropriate.”*

We agree that “over- or under-representation” is a more objective way of describing our

findings. We have substituted “strengthened” and “weakened” with “over-“ and “underrepresented”

in the text.

*4. The examples shown in Fig. 4 C and D are informative regarding the subset of MN specific*

*events with evidence for direct Nova regulation. It would also be useful if the authors could*

*indicate the calculated Percent Spliced I (PSI) for these exons to give a clearly picture as to*

*the degree of difference in splicing in each dataset. It would also be useful if the same*

*schematics could also be shown in a supplemental figure for the other 6 of these events.*

We have added dI (Delta Inclusion rate) in all Figures 4 and 5. dI is defined below1 (from

Ule et al., Nature Genet 2015); we find this to be a more reliable measure of splicing

differences than PSI as transcripts that have near zero inclusion under one condition, skewing

comparisons with distorted PSIs (dividing by near-zero) but yielding quantitatively reliable

dI’s. We have provided as Figure S5 the same schematics for the other six events where

MN-specific NOVA binding correlate with MN-specific alternative splicing.

*5. It would be helpful if the authors could describe or show the stoichiometry of Nova2*

*expression levels in MNs compared to endogenous Nova (beyond that in whole spinal lysates*

*shown in Fig. 1B). Are the levels similar to endogenous, lower, overexpressed? This would*

*be expected to impact the specificity as well as yield of the MN-specific CLIP observed.*

The reviewer raised an important point that we would also like to be able to address.

However, we currently do not have a good method to determine MN-specific transgene vs.

endogenous NOVA ratios, particularly within the entire MN compartment (ie FACS sorting

would lead to unacceptable loss of ~60% of the volume of a MN present in dendrites, and at

least some in the proximal axon). We therefore have used endogenous Nova in WSC as our

best means of estimating this variable.

**Response to Reviewer 2:**

*1. One should be careful about interpreting the results of alternative splicing analysis. The*

*authors compare MN CLIP with WSC CLIP and match it to alternative splicing analysis of*

*the MN and WSC RNA-Seq. Whereas motoneuron CLIP and MN RNA-Seq are indeed*

*matching datasets, WSC RNA-Seq comprises RNA from all cell types of the spinal cord,*

*neuronal and non-neuronal, whereas WSC CLIP only corresponds to the cell types that*

*express Nova2 (presumably, only neurons). This needs to be clarified and addressed properly.*

This is an excellent point. We acknowledge the limitation and added significant text in the

Discussion to address it. P25.

*2. The GO enrichment in terms "microtubule binding", "tubulin binding" and "cytoskeletal*

*protein binding" (Fig. 2E) may not be due to some of these targets being found specifically in*

*motoneurons. Rather, the total number of Nova2 targets identified in motoneurons (MN) was*

*less (173) than in the whole spinal cord (WSC) (301), and the depth of MN CLIP datasets is*

*several fold less than that of the WSC CLIP datasets. Cytoskeleton-related proteins tend to*

*be highly expressed and might have avoided under-detection relative to other targets. As*

*such, you cannot use these datasets for direct comparisons such has GO without further*

*normalization (eg subsampling). Do these target genes contain also quantitatively*

*strengthened MN peaks?*

We normalized WSC and MN CLIP signal per gene (CLIP reads in WSC or MN peaks per

gene normalized by read depth) by gene expression (rpkm in WSC or MN RNA-seq), and

calculated the ratio of MN vs. WSC (MN_CLIP * WSC_gene_expression/

(MN_gene_expression * WSC_CLIP)). This ratio is a quantitative estimate of strengthening

/weakening of MN vs. WSC CLIP signals per transcript copy. As shown below, cytoskeleton

interacting genes have significantly higher MN/WSC CLIP/RNA-seq ratios (p < 2.2 x 10-16, t

test), suggesting that these GO enriched NOVA target genes contain quantitatively

strengthened MN peaks. We have discussed this point on P20, and added the following figure

as Figure S8.

*3. The effect of knocking down Sept8-X1 isoform on the formation of dendritic spines is striking*

*and very specific. Therefore, it is indeed surprising that Nova2 knockdown has no*

*measurable effect on dendritic spine numbers. The authors discuss that this could be because*

*of either not sufficient effect of Nova2 knockdown or other targets of Nova2 possibly having*

*antagonistic effect on spine formation. An alternative explanation could be that in*

*hippocampal neurons, as opposed to motoneurons, Nova2 does not affect splicing of Sept8-*

*X1 isoform, and Nova2 KD effect on arborization is through other Nova2 targets (among*

*which are many cytoskeleton related proteins), which is partially mitigated by Sept8-X1*

*overexpression. Does Sept8-X1 overexpression by itself increase arborization? (for example,*

*in scrambled shRNA control?) Also, immunostaining of Sept-X1 and Nova2 KD hippocampal*

*neurons with X1-specific antibody could help to access the efficiency of Sept8-X1 isoform*

*depletion in this neuronal type.*

We overexpressed SEPT8-X1 in hippocampal cultures and performed Sholl analysis. SEPT8-

X1 overexpression does not affect dendritic complexity, as indicated by similar Sholl critical

values compared to neurons expression scramble shRNA (17.72 ± 1.96, n = 5 compared to

neurons expressing scramble shRNA: 18.23 ± 0.97, n = 17).

Since both SEPT8-X5 and SEPT8-X1 are expressed in hippocampal neurons, we would need

to use a SEPT8-X1 specific antibody for immunostaining. However, despite significant

effort, we could not identify any such an antibody that works well for immunofluorescence.

*4. For experimental reproducibility, more details need to be included on how exactly spinal*

*cords were processed for CLIP. Were they crosslinked as cell suspension? How was tissue*

*dissociation performed? Was the integrity of the cells microscopically checked during tissue*

*dissociation? Since the manuscript claims to have quantified Nova2 binding "in both the cell*

*bodies and processes", this needs to be clarified*

We updated the Methods section with a detailed procedure of spinal cord sample preparation

for HITS-CLIP. We would note that these were acutely dissected spinal cords, immediately

triturated, and UV-irradiated to crosslink protein-RNA complexes. We did not

microscopically check cell integrity. Although we expect disruption of cellular processes

from cell soma, we do not expect disruption of RNA-protein complexes during this brief prep

period (although we cannot entirely rule some effects of Heisenberg uncertainty principle

here), the processes are still present during the UV irradiation step, regardless of whether

they were attached to cell soma or disrupted. In that sense, integrity of the cells is not an

issue. We also have undertaken parallel CLIP experiments (not related to the current

manuscript) in the hippocampus, and found that CLIP from manual dissection of the soma

and cell processes yields target that in aggregate are the sum of both compartments. We

underscore that our RNA sequencing of NOVA MN CLIP’d complexes shows appropriate

specificity to NOVA YCAY binding targets. Altogether, we conclude that acute dissection

(and brief trituration, perhaps an unnecessary step given UV’s ability to penetrate tissue, but

done conservatively here) allows for recovery of intact crosslinked NOVA-RNA complexes

in neuronal processes.

*5. Also, please be more detailed in your computational analysis, eg peak calling. It shouldn't be*

*necessary for the reader to consult multiple manuscripts to consolidate "previously*

*described" approaches (I would be very much (positively) surprised if the data analysis*

*across all these previous studies was completely identical and thus unambiguous and*

*reproducible).*

We appreciate this comment, and have added the details about our bioinformatics pipeline in

Methods (P30). For peak calling, we ran our in-house algorithm with the exact parameters as

the quoted references.

*6. It is not very clear from the text that Nova2 protein apparently has two isoforms, longer and shorter (Western blot in Fig. 1B). Whereas GFP-Nova2 construct and thus motoneuron (MN) CLIP was made with the short isoform, it is not explicitly mentioned with which isoform(s) the whole spinal cord (WSC) CLIP on endogenous Nova2 was done. It appears from the radioactive gel in Fig. S2A that it was the long isoform (cf. Fig. 1B), but a clarification, and a discussion why which isoform was chosen, would be helpful.*

We have previously performed CLIP experiments using isoform-specific NOVA2 antibodies for the long and short NOVA2 proteins (which are nearly identical, including identical RNA binding domains), and detect essentially identical RNA binding targets in the long and short NOVA2 isoforms (Yano Y, unpublished data). We chose to epitope tag the longer NOVA2 isoform to further minimize potential contamination from co-purified RNA-protein complexes, considering that most RNA-binding proteins are below 60 KD. We have clarified these points on P4, 5 & 7.

*7. Gene names such as Sept8 and March1 have been automatically converted to date by Excel in supplementary tables (e.g. Additional File 1, Sup_1B).*

We appreciate this observation (!) and have formatted the gene symbol columns in all Additional Files to “text” to prevent auto correction.

*8. Vertical range scale for coverage is missing on the figures that contain genome browser screenshots (Fig. 4C,D, 5C). (- Many relative MN peak heights as in Fig. 3A,B seem to show discrete values. That possibly might arise from joined peaks that contain low MN read numbers but are being called due to sufficient reads present in the WSC dataset. It might be beneficial to increase stringency for filtering on minimal number of reads coming from each respective sample.)*

Vertical ranges in unit of reads per million have been added to all genome browser

figures to indicate CLIP and RNA-seq read coverage.

We introduced Joint Peaks to facilitate comparison between NOVA binding in WSC

and MN, in that Joint Peaks denote common genomic coordinates of NOVA binding

sites in WSC and/or MN. Therefore, Joint Peaks are designed to be inclusive of

NOVA binding sites that are exclusively in WSC, exclusively in MN, and in both

WSC and MN as mentioned by the reviewer. For comparison between WSC and MN

NOVA binding, peaks with only WSC or MN CLIP reads need to be included. On the

other hand, to define NOVA binding sites in WSC or MN, we called CLIP peaks using

only WSC or MN CLIP dataset, as shown in Figure 2.

The discrete values in Figure 3A and B arise in many cases from genes with fewer

MN CLIP raw reads . Since less overall CLIP reads were obtained for MN CLIP, we

expect more transcripts to have fewer MN raw CLIP reads, and therefore more peaks

with coincided MN relative peak heights (e.g, a peak of 5 MN CLIP raw reads in a

gene with 10 MN CLIP raw reads has the same relative peak height as a peak of 6 MN

CLIP reads in a gene with 12 MN CLIP raw reads).

9. *The discussion focuses on a phenotype of Nova1/Nova2 double knockout mouse but does not address a possible degree of mutual compensation between these RBPs. A discussion of motoneuron phenotype specifically of Nova2 knockout mouse in the context of the MNNova2-targetome is missing.*

Phenotype of the Nova1/Nova2 double knockout mouse is indeed most relevant due to known NOVA1-NOVA2 functional compensation (Ruggiu, PNAS, 2009). NOVA1 and NOVA2 proteins are both expressed in spinal motor neurons and show nearly identical RNAbinding profiles in WSC. Conditionally deleting either NOVA1 or NOVA2 in motor neurons produced no phenotype, yet deleting both NOVA1 and NOVA2 was synthetically lethal (Yuan Y, unpublished data), reflecting the lethal motor paralysis phenotype of complete NOVA1/NOVA2 double KO mice (Ruggiu, PNAS, 2009). We discuss the motor phenotype in the Nova1/Nova2 double knockout in this context.

**Second round of review**

**Reviewer 1**

**Please provide your comments for the author(s) on the revised manuscript.**

The Author's have addressed my critiques. Only minor point is noted p19, line 14 should be "understanding" not "understand."

**Evaluation. Has the author satisfactorily responded to your previous review?**

Yes
